# eTFC-01: a dual-labeled chelate-bridged tracer for SSTR2-positive tumors

**DOI:** 10.1186/s41181-024-00272-0

**Published:** 2024-05-22

**Authors:** Dylan Chapeau, Savanne Beekman, Maryana Handula, Erika Murce, Corrina de Ridder, Debra Stuurman, Yann Seimbille

**Affiliations:** 1 Erasmus MC, Department of Radiology and Nuclear Medicine, University Medical Center Rotterdam, Rotterdam, The Netherlands; 2 Erasmus MC Cancer Institute, Rotterdam, The Netherlands; 3 TRIUMF, Life Sciences Division, Vancouver, Canada

**Keywords:** NETs, SSTR2, Dual-labeled tracer, Nuclear medicine, Fluorescence-guided surgery

## Abstract

**Background:**

Integrating radioactive and optical imaging techniques can facilitate the prognosis and surgical guidance for cancer patients. Using a single dual-labeled tracer ensures consistency in both imaging modalities. However, developing such molecule is challenging due to the need to preserve the biochemical properties of the tracer while introducing bulky labeling moieties. In our study, we designed a trifunctional chelate that facilitates the coupling of the targeting vector and fluorescent dye at opposite sites to avoid undesired steric hindrance effects. The synthesis of the trifunctional chelate N_3_-Py-DOTAGA-(tBu)_3_ (**7**) involved a five-step synthetic route, followed by conjugation to the linear peptidyl-resin **8** through solid-phase synthesis. After deprotection and cyclization, the near-infrared fluorescent dye sulfo-Cy.5 was introduced using copper free click chemistry, resulting in **eTFC-01**. Subsequently, **eTFC-01** was labeled with [^111^In]InCl_3_. In vitro assessments of eTFC-01 binding, uptake, and internalization were conducted in SSTR2-transfected U2OS cells. Ex-vivo biodistribution and fluorescence imaging were performed in H69-tumor bearing mice.

**Results:**

**eTFC-01** demonstrated a two-fold higher IC50 value for SSTR2 compared to the gold standard DOTA-TATE. Labeling of **eTFC-01** with [^111^In]InCl_3_ gave a high radiochemical yield and purity. The uptake of [^111^In]In-**eTFC-01** in U2OS.SSTR2 cells was two-fold lower than the uptake of [^111^In]In-DOTA-TATE, consistent with the binding affinity. Tumor uptake in H69-xenografted mice was lower for [^111^In]In-**eTFC-01** at all-time points compared to [^111^In]In-DOTA-TATE. Prolonged blood circulation led to increased accumulation of [^111^In]In-**eTFC-01** in highly vascularized tissues, such as lungs, skin, and heart. Fluorescence measurements in different organs correlated with the radioactive signal distribution.

**Conclusion:**

The successful synthesis and coupling of the trifunctional chelate to the peptide and fluorescent dye support the potential of this synthetic approach to generate dual labeled tracers. While promising in vitro, the in vivo results obtained with [^111^In]In-**eTFC-01** suggest the need for adjustments to enhance tracer distribution.

**Supplementary Information:**

The online version contains supplementary material available at 10.1186/s41181-024-00272-0.

## Introduction

Surgery remains the primary treatment for the majority of solid tumors. However, the challenge arises from the positive surgical margins, leading to the possibility of missing residual tumors cells. This oversight can contribute to local recurrence and, consequently a decline in overall patient survival(Orosco et al. 2018). To overcome this challenge, innovative techniques have been developed to assist surgeons during operations, such as radio-guided surgery (RGS). RGS used γ-photons emitted by a radiotracer and a portative γ-camera(Gulec and Baum 2007; Valdés Olmos et al. 2009). However, radioguidance suffers from relatively poor spatial resolution, which is limiting visual discrimination between tumor and healthy tissues and increases the risk of incomplete resection(Hussain and Nguyen 2014; Lütje et al. 2014). Unlike RGS, fluorescence-guided surgery (FGS) involves the injection of a fluorescent tracer to illuminate malignant tissues detectable with a specific camera(Nagaya et al. 2017; Sutton et al. 2023; Brookes et al. 2021). To conduct FGS, near-Infrared dyes are employed to prevent interference from tissue autofluorescence (600 nm) and water absorbance (> 900 nm)(Cornelissen et al. 2018; Themelis et al. 2009; Izzetoglu et al. 2005). The indocyanine green (ICG) and methylene blue (MB) are both NIRF dye approved by the Food and Drugs Agency (FDA) and are currently used in clinic(Schaafsma et al. 2011; Han et al. 2007). Carbocyanine dyes, such as Cyanine5 (Cy5) and IRDye 800CW, are among the most commonly used NIRF dyes in preclinical studies(Gorka et al. 2015). Sulfonated cyanine derivatives, such as Sulfo-Cy5, were developed to improve water solubility, chemical stability and the photophysical proprieties of the dyes(Buckle et al. 2018). Despite the emergence of this surgical technique, some limitations persist. A major drawback is the limited signal depth (1–2 cm, depending on the dye)(Zhu and Sevick-Muraca 2015; Chin et al. 2012; Leeuwen et al. 2017), allowing visualization only at the tissue surface. To overcome this limitation, optical imaging can be combined with other imaging modalities, such as positron emission tomography (PET) and single-photon emission computed tomography (SPECT). This combination enables patients to undergo a non-invasive whole-body nuclear scan to quantitatively assess the radioactive agent’s distribution. Subsequently, the near-infrared fluorescence (NIRF) signal facilitates the visualization and precise resection of the neoplastic lesions, as well as ex vivo histopathology analyses to confirm the presence of malignant cells. The rationale behind using a single dual-labeled tracer, instead of a radioactive tracer for preoperative prognosis and a fluorescent tracer for surgical guidance, is that their distribution may differ due to the distinct chemical and physical properties of these two tracers. The development of a dual-labeled compound is complex, but it is essential for the successful integration of both imaging techniques, since distribution of the tracer remains unchanged between the nuclear images and FGS(Jennings and Long 2009).

In this study, we selected neuroendocrine tumors (NETs) as the model system to validate our approach for the development of a dual-labeled tracer. Imaging of NETs plays an important role in the detection of primary tumors, metastatic tissues and guiding treatment decisions(Maxwell and Howe 2015; Essen et al. 2014). Notably, 70% of NETs patients(Chakedis et al. 2019), particularly gastroenteropancreatic neuroendocrine tumors (GEP-NETs), necessitate surgery not only for primary tumor removal but also to avoid hormone production from metastatic tissues (Partelli et al. 2014; Birnbaum et al. 2015) and improved the overall survival(Menda et al. 2013). Existing radiolabeled peptides, such as [^68^Ga]Ga-DOTA-TATE or [^64^Cu]Cu-DOTA-TATE, that target the somatostatin receptor subtype 2 (SSTR2) receptor are already established in clinical settings for preoperative imaging of NETs(Menda et al. 2013; Kroiss et al. 2015; Pfeifer et al. 2012). While radiation-guided surgery has been used for NETs, the contrast for tumor visualization remains low, leading to partial resections(Cockburn et al. 2021; Adams et al. 1998). This dual-modality strategy aims to enhance the overall precision of detection and removal of NETs(Achilefu et al. 2002). Multiple dual-labeled scaffolds have previously been reported in the literature(Barry Edwards et al. 2009; Kuil et al. 2011; Handula et al. 2022a, b; Wang et al. 2020). The majority of the dual-labeled tracers are based on the use of a linker, usually a lysine residue, to attach the chelate and the dye to the targeting vector. Santini et al. developed a SSTR2-targeted tracer based on this strategy and showed that it allowed nuclear and fluorescence detection of the SSTR2-positive tumor. However, it was also reported that the biological proprieties (e.g., affinity, uptake/internalization, biodistribution) were altered(Santini et al. 2016). Other approaches employed either the chelate or the dye as a linker to attach the targeting vector with the complementary label. Ghosh et al. (Ghosh et al. 2017a, b). and Licha et al.(Heing-Becker et al. 2021) developed SSTR2-targeted tracers using the chelate or the dye as linkage, respectively. In both approaches, the in vitro and in vivo results were very promising, showing a retention of the pharmacological properties of the parent peptide. However, recent review raised the chemical challenge of those strategies(Ariztia et al. 2022; Munch et al. 2020) and none of them have been implemented into clinics yet.

In our study, we opted for octreotate (TATE) as targeting vector for NETs. This choice was driven by its selective targeting of SSTR2, which is prominently expressed in the neuroendocrine tumor cells and its application in clinics(Cwikla et al. 2009; Kwekkeboom et al. 2008; Strosberg et al. 2017). In an effort to induce minimal alterations to the binding and pharmacokinetic properties of the gold standard DOTA-TATE, we synthetized a trifunctional chelate (N_3_-Py-DOTAGA) based on the macrocyclic 1,4,7,10-tetraazacyclododecane-1,7-diacetic acid (DO2A) chelate. N_3_-Py-DOTAGA was designed to enable site-specific labelling with a fluorescent dye and mitigate steric effects. Subsequently, we coupled this chelate to both the TATE peptide and Sulfo-Cy5. Finally, we performed a comprehensive comparison of the in vitro and in vivo characteristics of our dual-labeled tracer, **eTFC-01**, with those of DOTA-TATE.

## Methods

### Chemistry

All chemicals and solvents were obtained from commercial suppliers and used without further purification, unless specified. DO2A-*tert*-butyl ester and 2-chlorotrityl chloride resin were purchased from Chematech (Dijon, France) and Advanced Chemtech (Louisville, KY, USA), respectively. The peptide sequence was synthesized manually using standard solid phase synthesis protocols. ^1^H NMR spectra were recorded at 600 MHz on Bruker AMX600 spectrometers (Delft, The Netherlands) and ^13^C NMR at 15 MHz on Nanalysis 60PRO (Calgary, Canada) at ambient temperature in CDCl_3_ unless specified. The chemical shifts (δ) for ^1^H and ^13^C are quoted relative to residual signals of the solvent on the ppm scale. Coupling constants (*J* values) are reported in Hertz (Hz) and are H-H coupling constants unless otherwise stated. Quality control was performed by LC-MS using an Agilent 1260 Infinity II LC/MSD XT system (Amstelveen, The Netherlands). Electrospray ionization in positive mode was used to confirm the identity of the obtained products. Purification of the synthesized ligands were performed by preparative HPLC on an Agilent 1290 Infinity II or by semi-preparative HPLC on a Waters 2695 system (Etten-Leur, The Netherlands) equipped with a diode array detector 2998. The LC-MS and the HPLC were controlled by Agilent OpenLab CDS Chemstation or Empower 3 software, respectively.

### Radiochemistry

[^111^In]InCl_3_ was ordered from Curium (Petten, The Netherlands). Quality control was performed by high performance liquid chromatography (HPLC) using a Waters 2695 system (Etten-Leur, The Netherlands) equipped with a diode array detector 2998 and a radioactivity detector from Canberra (Zadik, Belgium). The HPLC was controlled by Empower3. Instant thin-layer chromatography plates (iTLC) were analyzed by a bSCAN radio-chromatography scanner from Brightspec (Antwerp, Belgium) equipped with a sodium iodide detector. Activity measurements were performed using a VDC-405 dose calibrator from Comecer (Joure, The Netherlands). The radioactive samples used for the determination of LogD_7.4_, in vitro assays, and in vivo uptake in tissues were counted using a Wizard 2480 gamma counter from Perkin Elmer (Waltham, MA, USA).

### HPLC conditions

The LC-MS analyses were performed on a Poroshell 120 EC-C18 column (3 × 100 mm, 2.7 μm) at a flow rate of 0.5 mL/min and with a mobile phase consisting of: A (0.1% formic acid in water (*v/v*)) and B (0.1% formic acid in acetonitrile (*v/v*)). Elution of the products was performed according to the following gradient: 0–5 min, 5–100% B; 5–8 min, 100% B.

Purification of compounds **1–7** was performed using an Agilent 5 Prep C18 column (50 × 21.2 mm, 5 μm). The mobile phase consisted of solvents A (0.1% Trifluoroacetic acid (TFA)in water (*v/v*)) and B (0.1% TFA in acetonitrile (*v/v*)) at a flow rate of 10 mL/min. The following elution method was used: 0–8 min: 5–100% B and 8–10 min: 100% B.

Purification of peptides **9–11** was performed using a semi-preparative Luna RP-C_18_ column (10 μm, 250 × 10 mm) from Phenomenex (Le Pecq, France) at a flow rate of 3 mL/min with either Condition 1 (a gradient of acetonitrile (10–95%) in water containing 0.1% TFA over 20 min) or Condition 2 (an isocratic elution with 27% acetonitrile in water containing 0.1% TFA).

The analysis of the radioactive products was performed by radio-HPLC on an analytical Gemini C_18_ column (5 μm, 250.0 × 4.6 mm) from Phenomenex at a flow rate of 1 mL/min and with a mobile phase consisting of: A (0.1% TFA in water (*v/v*)) and B (0.1% TFA in acetonitrile (*v/v*)). Elution of the products was performed according to the following gradient: 0–3 min: 5% B; 3–23 min: 5 to 100% B and 23–27 min: 100% B.

### Synthetic method

#### 2-((3-azidopropoxy)methyl)-6-(bromomethyl)pyridine (2)

To a solution of 3-azidopropanol (3.18 g; 31.5 mmol) in dry THF (200 mL) and under a nitrogen atmosphere was added 2,6-bis(bromomethyl)pyridine (10 g, 37.7 mmol; 1.2 equiv.) and potassium tert-butoxide (7.04 g, 62.8 mmol; 2 equiv.) were added. The resulting mixture was stirred overnight at room temperature. The solvent was then evaporated under reduced pressure. The residue was purified by flash chromatography column using ethyl acetate/hexane as solvent (from 1:9 to 1:4 v/v) to give 1.39 g of 2 as an orange oil (18%). 1H NMR (600 MHz, Chloroform-d) δ 7.88–7.16 (m, 3H), 4.59 (s, 2H), 4.50 (s, 2H), 3.52 (m, 4H), 1.89 (p, J = 6.0 Hz, 2H); 13 C NMR (15 MHz, Chloroform-d) δ 137.89, 122.33, 120.72, 77.16, 75.02, 67.83, 46.52, 32.29, 29.48. ESI-MS: m/z calc’ for C10H13BrN4O 284.04; found 285.00 [M + H]+.

#### 5-benzyl-1-***tert***-butyl 2-bromopentanedioate (4)

2-Amino-5-ethyl-5-oxapentanoic acid (6 g; 34.53 mmol) and potassium bromide (17 g; 143 mmol; 4 equiv.) were dissolved in a solution of 1 M aqueous hydrobromic acid (70 mL; 70 mmol; 2 equiv.). The resulting solution was cooled to 0 °C, and sodium nitrite (4.76 g; 69.02 mmol; 2 equiv.) was added portion-wise. Then, the mixture was stirred for 3 h at room temperature. Concentrated sulfuric acid (2 mL) was added into the mixture and the product was extracted thrice with diethyl ether. The combined organic phases were washed with water, dried over anhydrous MgSO4 and then concentrated under reduce pressure. The residue was dissolved in DCM (100 mL). A solution of tert-butyl-2,2,2-trichloroacetimidate (15.30 g; 70 mmol; 2 equiv.) in cyclohexane (100 mL) was added, followed by dimethylacetamide (3.1 mL; 69.09 mmol; 2 equiv.). Then, boron trifluoride etherate (1.7 mL; 13.80 mmol; 0.4 equiv.) was added dropwise. The mixture was stirred at room temperature overnight. After evaporation of the solvent, the residue was dissolved in hexane and the suspension was filtrated and washed twice with hexane. The filtrate was then concentrated under reduced pressure, and the crude product was purified by column chromatography using hexane/ethyl acetate (4:1, v/v) to give 2.81 g of 4, as a colorless liquid (28%). 1 H NMR (60 MHz, Chloroform-d) δ 4.22–3.73 (m, 3 H), 2.43–1.95 (m, 4 H), 1.29 (s, 9 H), 1.07 (t, J = 7.1 Hz, 3 H). 13 C NMR (15 MHz, Chloroform-d) δ 171.34, 167.73, 82.28, 60.04, 46.30, 31.18, 29.55, 27.32, 13.86.

#### 1-(***tert***-Butyl) 5-ethyl 2-(4,10-bis(2-(***tert***-butoxy)-2-oxoethyl)-1,4,7,10-tetraazacyclododecan-1-yl)pentanedioate (5)

To a solution of DO2A-tert-butyl ester (2.57 g; 6.4 mmol; 1.5 equiv.) in acetone (200 mL), potassium carbonate (0,89 g; 6,4 mmol; 1,5 equiv.) and potassium iodide (0.21 g; 1.3 mmol; 0.3 equiv.) were added. The mixture was stirred for 10 minutes. Then, a solution of 4 (1.28 g; 4.3 mmol) in acetone (50 mL) was added dropwise. The reaction mixture was refluxed overnight. Afterwards, salts were removed by filtration. The solution was concentrated, and the residue was purified by flash chromatography using DCM/MeOH (99:1 to 95:5 v/v) to give 1.24 g of 3 as a yellow-orange oil (47%). 1H NMR (600 MHz, Chloroform-d) δ 9.32 (s, 1H), 4.17–4.04 (m, 2H), 3.49–3.28 (m, 5H), 3.23–3.02 (m, 7H), 2.81 (m, 5H), 2.57–2.47 (m, 2H), 2.47–2.34 (m, 2H), 1.93 (q, J = 6.8 Hz, 2H), 1.43 (s, 9H), 1.42 (s, 18H), 1.26–1.19 (m, 3H). 13 C NMR (15 MHz, Chloroform-d) δ 161.83, 81.60, 79.14, 77.02, 74.91, 60.64, 56.45, 53.50, 51.63, 50.71, 49.67, 46.40, 28.13, 14.22. ESI-MS: m/z calc’ for C31H58N4O8 614.83; found 615.40 [M + H]+.

#### 1-(***tert***-Butyl) 5-ethyl 2-(7-((6-((3-azidopropoxy)methyl)pyridine-2-yl)methyl)-4,10-bis(2-(***tert***-butoxy)-2-oxoethyl)-1,4,7,10-tetraazacyclododecan-1-yl)pentanedioate (6)

To a solution of 5 (229 mg; 0.37 mmol) in ACN (50 mL), potassium phosphate (158 mg; 0.74 mmol; 2 equiv.) and potassium iodide (19 mg; 0.11 mmol; 0.3 equiv.) were added. The mixture was stirred for 10 minutes. Then, a solution of 2 (106 mg; 0.37 mmol; 1 equiv.) in ACN (5 mL) was added dropwise. The reaction mixture was refluxed overnight. Then, salts were removed by filtration. The solution was concentrated, and the residue was purified by preparative HPLC to give 119 mg of 6 a yellow oil (39%). ^1^H NMR (600 MHz, Chloroform-*d*) δ 9.19 (s, 1H), 7.76 (t, *J* = 7.7 Hz, 1H), 7.45 (d, *J* = 7.8 Hz, 1H), 7.39 (d, *J* = 7.6 Hz, 1H), 4.58 (s, 2H), 4.55 (d, *J* = 14.4 Hz, 1H), 4.36 (d, *J* = 14.3 Hz, 1H), 4.13–4.06 (m, 2H), 3.68 (s, 2H), 3.62 (t, *J* = 6.0 Hz, 2H), 3.61–3.46 (m, 4H), 3.47–3.39 (m, 4H), 3.31–3.28 (m, 1H), 3.17–2.96 (m, 9H), 2.52–2.48 (m, 2H), 2.06–1.96 (m, 2H), 1.90 (p, *J* = 6.3 Hz, 2H), 1.49 (s, 9H), 1.41 (s, 18H), 1.22 (t, *J* = 7.1 Hz, 3H). ^13^C NMR (15 MHz, Chloroform-*d*) δ 169.33, 158.88, 139.01, 124.11, 82.94, 73.32, 69.29, 67.93, 62.59, 61.02, 55.79, 50.47, 50.04, 48.34, 30.82, 29.34, 28.09, 14.41. ESI-MS: m/z calc’ for C_41_H_70_N_8_O_9_ 818.52; found 819.5 [M + H]^+^.

#### 4-(7-((6-((3-azidopropoxy)methyl)pyridin-2-yl)methyl)-4,10-bis(2-(***tert***-butoxy)-2-oxoethyl)-1,4,7,10-tetraazacyclo dodecan-1-yl)-5-(***tert***-butoxy)-5-oxopentanoic acid (7; N_3_-Py-DOTAGA-(***t***Bu)_3_)

6 (119 mg; 0.15 mmol; 1 equiv.) was dissolved in 1,4-dioxane (5 mL). Then, 1 M NaOH was added dropwise until the pH reached approximately 8.5. The reaction was monitored by LC-MS. The reaction mixture was then stirred at room temperature until complete deprotection was achieved. The mixture was concentrated and the residue was purified using preparative HPLC to give 67 mg of compound 7, as a colorless oil (58%). ^1^H NMR (600 MHz, Chloroform-*d*) δ 7.90–7.46 (m, 3H), 4.63 (s, 3H), 3.92–3.62 (m, 5H), 3.63–3.55 (m, 3H), 3.38 (t, *J* = 6.5 Hz, 3H), 3.33–2.77 (m, 8H), 2.66 (d, *J* = 50.1 Hz, 2H), 2.15–1.94 (m, 1H), 1.86 (p, *J* = 6.2 Hz, 3H), 1.43 (d, *J* = 85.1 Hz, 27H). ^13^C NMR (15 MHz, CDCl_3_) δ 174.97, 160.99, 160.74, 160.50, 160.25, 118.79, 116.87, 114.95, 113.03, 77.37, 77.16, 76.95, 67.98, 60.46, 59.59, 55.09, 53.50, 51.49, 48.31, 30.73, 29.69, 29.00, 27.78, 27.64, 27.20, 20.34. ESI-MS: m/z calc’ for C_39_H_66_N_8_O_9_ 790.45; found 791.4 [M + H]^+^.

#### N_3_-Py-DOTAGA-D-Phe-Cys-Tyr-D-Trp-Lys-Thr-Cys-Thr-OH (9)

D-Phe-Cys(Trt)-Tyr(*t*Bu)-D-Trp(Boc)-Lys(Boc)-Thr(*t*Bu)-Cys(Trt)-Thr(*t*Bu)-resin was synthesized using a N𝛼-Fmoc solid-phase peptide synthesis (SPPS) strategy. The conjugation of Fmoc-protected amino acid (4.0 equiv.) to the 2-chlorotrityl chloride resin was carried out in dimethylformamide (DMF) using hexafluorophosphate azabenzotriazole tetramethyl uronium (HATU; 3.8 equiv.) and *N*,*N*-diisopropylethylamine (DIPEA; 7.8 equiv.) for 45 min. Fmoc deprotection was accomplished by treating the resin with a 20% solution of piperidine in DMF for 15 min. Amide formation and Fmoc deprotection were monitored by the Kaiser test. Coupling and deprotection were performed twice when the reaction was not completed. Peptide synthesis started by loading Fmoc-L-Thr(*t*Bu)-OH onto the 2-chlorotrityl chloride resin (150 mg, average loading capacity: 0.6 mmol/g). The resin was shaken for 90 min at room temperature. The resin was capped using 1 mL of dichloromethane/methanol/DIPEA (80:15:5 v/v/v) for 15 min at rt. Subsequent Fmoc deprotection and coupling with Fmoc-L-Cys(Trt)-OH, Fmoc-L-Thr(*t*Bu)-OH, Fmoc-L-Lys(Boc)-OH, Fmoc-D-Trp(Boc)-OH, Fmoc-L-Tyr(*t*Bu)-OH, Fmoc-L-Cys(Trt)-OH, and Fmoc-D-Phe-OH were achieved following the protocols described above. After the ultimate Fmoc deprotection, **7** (1.5 equiv.), HATU (1.5 equiv.), and DIPEA (10.0 equiv.) in 1 mL of DMF were added to the resin, and the mixture was stirred overnight at rt. Conjugation of the chelator was confirmed by analytical HPLC after cleavage and deprotection of a small sample of peptide. After completion of the reaction, the resin was washed with DMF (5 × 1 mL) and dichloromethane (3 × 1 mL). A solution of TFA/H_2_O/TIPS (95:2.5:2.5 v/v/v) was added to the resin, and the mixture was stirred for 3 h at rt. The solution was removed from the resin by filtration, and ice-cold diethyl ether was added to the filtrate. The precipitate, collected by centrifugation, was dissolved in neat TFA and the mixture was stirred for 48 h at room temperature. TFA was removed by a gentle airflow and the peptide was precipitated by adding cold diethyl ether and collected after centrifugation. The crude product was purified by semi-preparative HPLC to give 10 mg of **9** as a white solid (6%). ESI-MS: m/z calc’ for C_76_H_106_N_18_O_20_S_2_ 1655.91; found 1656.50 [M + H]^+^.

#### N_3_-Py-DOTAGA-D-Phe-cyclo[Cys-Tyr-D-Trp-Lys-Thr-Cys]-Thr-OH (10)

The linear peptide **9** (4.5 mg; 0.483 μmol) was dissolved in 100 mM monosodium phosphate buffer (pH = 7) to achieve a concentration of 1 mg/mL. Then, a solution of 5% DMSO in 2 mM Gdn.HCl buffer was added into the mixture. The resulting mixture was stirred at room temperature for 48 h. The solution was concentrated under high pressure. Subsequently, the residue was purified by semi-preparative HPLC to give 2.8 mg of **10** as a white solid (63%). ESI-MS: m/z calc’ for C_76_H_104_N_18_O_20_S_2_ 1653.89; found 1654.60 [M + H]^+^.

#### eTFC-01 (11)

**10** (1.1 mg; 0.665 μmol) and sulfo-Cyanine5 DBCO (1 mg; 1.02 μmol; 1.5 equiv.) were dissolved in H_2_O/ACN (1 mL; 1:1 v/v). The mixture was stirred for 1 h at 37°C. Then, the mixture was directly purified by semi-preparative HPLC to give 1,6 mg of **eTFC-01** as a blue solid (94%). Purity > 99%. ESI-MS: m/z calc’ for C_129_H_161_N_22_O_28_S_4_ 2596.07; found 1299.30 [M + 2 H]^2+^.

### Radiolabeling with [^111^In]InCl_3_

Concentration of the peptide precursor was determined via titration according to a method previously reported (Handula et al. 2022a, b). [^111^In]InCl_3_ (20 MBq) in 0.5 M HCl was added to a mixture of DOTA-TATE or **eTFC-01** (1 nmol), ascorbic acid/gentisic acid (10 μL, 50 mM), ethanol (10 μL), sodium acetate (1 μL, 2.5 M) and H_2_O (final volume of 140 μL). The mixture was incubated for 20 min at 90 °C. The reaction was monitored by iTLC on silica gel impregnated glass fiber plates iTLC-SG (Agilent; Amsterdam, the Netherlands) eluted with a solution of sodium citrate (0.1 M, pH 5.0). Diethylenetriaminepentaacetic acid (DTPA) (5 μL, 3 mg/mL) was added to complex the remaining free indium-111. An aliquot was taken and injected into radio-HPLC.

### In vitro stability studies

[^111^In]In-**eTFC-01** (∼ 1 MBq) was incubated in 300 μL of phosphate buffered saline (PBS; 0.1 M, pH 7.4) or mouse serum at 37 °C. Stability of the radiolabeled peptide was monitored at 1, 4 and 24 h post incubation. The samples in PBS were directly analyzed by radio-HPLC without any pretreatment. However, samples in mouse serum were mixed with an equal volume of acetonitrile to precipitate the proteins. The solution was centrifuged for 10 min at 13,000 rpm and the supernatant was analyzed by radio-HPLC.

### Distribution coefficient (LogD_7.4_)

Distribution coefficient was determined by a shake-flask method. A sample of [^111^In]In-**eTFC-01** (∼ 0.5 MBq) was added to a vial containing PBS (300 μL, pH 7.4) and n-octanol (300 μL). The vial was vortexed vigorously and then centrifuged at 10,000 rpm for 10 min for phase separation. Samples (100 μL) of the n-octanol and aqueous phases were taken and measured in the gamma counter. LogD_7.4_ value was calculated by using the following equation: LogD_7.4_ = log [(counts in n-octanol phase)/(counts in aqueous phase)]. Measurements were performed in triplicates.

### Cell culture

NCI-H69 cells (ATCC, Manassas, VA, USA) were cultured in Rosewell Park Medium Institute 1638 medium (RPMI-1638) (Sigma-Aldrich; Darmstadt, Germany) supplemented with penicillin (50 units/mL), streptomycin (50 μg/mL) and 10% fetal bovine serum (FBS). Cells were cultured at 37 °C and 5% CO_2_. Human osteosarcoma cells (U2OS) transfected with SSTR2 receptors were cultured in Dulbecco’s modified Eagle’s medium (DMEM) from Gibco (Paisley, UK) supplemented with 2 mM L-glutamine, 10% fetal bovine serum (FBS), 50 units/mL penicillin, and 50 μg/mL streptomycin. Cells were maintained at 37 °C and in a 5% CO_2_ humidified chamber. Passages were performed weekly using trypsin/EDTA (0.05%/0.02% w/v).

### Binding affinity assay

Competitive binding experiments against [^111^In]In-DOTA-TATE were performed with **eTFC-01** and DOTA-TATE in U2OS.SSTR2 cells. Cells were seeded in a 24-well plate 24 h prior the assay (2 × 10^5^ cells/well). Medium was removed and the cells were washed once with PBS. Then, solutions (10^− 12^ to 10^− 5^ M) containing unlabeled **eTFC-01** and DOTA-TATE in internalization medium (DMEM media, 20 mM HEPES, 1% BSA, pH 7.4) were added, followed by [^111^In]In-DOTA-TATE (10^− 9^ M). For each concentration, experiments were performed in triplicate. Cells were incubated at 37 °C for 90 min. After incubation, medium was removed and cells were washed twice with PBS and lysed with 1 M NaOH for 5 min at rt. The lysate was transferred to counting tubes, and measurement was performed using the gamma counter. The 50% inhibition constant (IC_50_) was calculated by fitting the data to a onesite Fit-Ki curve in GraphPad Prism v9.

### Uptake/internalization studies

Cells were seeded in 6-well plates 24 h before the experiment (7 × 10^5^ cells/well). The following day, adhered cells were incubated with 10^− 9^ M of [^111^In]In-DOTA-TATE or [^111^In]In-**eTFC-01** in 1 mL of culture medium for 90 min at 37 °C. Both compounds were also evaluated with a block of 50-fold excess non-labelled DOTA-TATE. The membrane-bound fraction was collected by incubating cells with an acid buffer (50 mM glycine, 100 mM NaCl, pH 2.8) for 10 min at rt. The internalized fraction was determined by lysing the cells with 1 M NaOH for 5 min at rt. Both fractions were measured in a gamma counter, and data were analyzed in GraphPad Prism v9 and expressed as percentage of added dose.

### Animal model

Six-week‐old female Balb/c nu/nu‐specific mice (Janvier Labs, Le Genest‐Saint‐Isle, France) were housed in individually ventilated cages (4 mice/cage). Upon arrival, mice were acclimated for one week, with access to food and water ad libitum. Mice were subcutaneously inoculated on the right shoulder with NCI-H69 cells (5 × 10^6^ cells) suspended in 100 μL of 1/3 Matrigel (Corning Inc.; Corning, NY, USA) and 2/3 Hank’s balanced salt solution (Gibco; Paisley, UK). NCI-H69 xenografts were allowed to grow for 2 weeks. Tumor size was 188 ± 60 mm^3^ at the start of the studies.

### Biodistribution studies

Biodistribution studies were performed to determine tumor and organ uptake of [^111^In]In-DOTA-TATE and [^111^In]In-**eTFC-01**. Mice were intravenously injected in the tail vein with 100 μL of [^111^In]In-DOTA-TATE (3.87 ± 0.15 MBq, 0.5 nmol) or [^111^In]In-**eTFC-01** (3.90 ± 0.19 MBq, 2 nmol) containing Kolliphor HS15 (Merck; Haarlerbergweg, The Netherlands) in PBS (0.06 mg/mL). At 3 selected time points (1, 4 and 24 h; *n* = 4) p.i., blood was collected via retro-orbital puncture under isoflurane/O_2_ anesthesia, after which the mice were sacrificed via cervical dislocation. The tumor and organs of interest (heart, lungs, liver, spleen, stomach, large intestine, small intestine, pancreas, kidneys, muscle, skin, bone) were excised, washed in PBS and blotted dry. To confirm receptor specificity of radioligands uptake, mice (*n* = 4) were co-injected with an excess (50 nmol) of DOTA-TATE and uptakes were determined at 4 h p.i. To determine the total injected radioactivity per animal, a calibration curve with indium-111 was established using the gamma counter. The percentage of injected dose per gram (% ID/g) was determined for each tissue and corrected for both the injected activity and % ID present at the injection site (the tail). Then, the tumor and the organs were placed in a petri dish and ex vivo optical imaging was performed with the IVIS Spectrum system (Perkin Elmer, Waltham, MA, USA) using the following settings for all measurements: FOV 19.5 cm, medium binning, f2, 60 s exposure with an excitation/emission filter of 646 nm/662 nm. Living Image version 4.5.2 software (Perkin Elmer) was used to perform data analysis by drawing regions of interest around the organ/tissue to quantify the radiant efficiency {(photons/second/cm^2^/steradian)/(μW/cm^2^)}.

### Statistical analysis

Statistical analysis and nonlinear regression were performed using GraphPad Prism 5 (GraphPad software, San Diego, CA, USA), and a Grubbs’ test (α = 0.05) was used to compare medians between groups. The Akaike information criterion was used to decide if a residual plateau needs to be included in the exponential fit. Data were reported as mean ± SEM (standard error of mean) for at least three independent replicates. The data sets were analyzed for significance using the one-way ANOVA using SigmaPlot 15.0 software. A *P*-value lower than 0.05 was considered statistically significant.

## Results

### Chemistry and radiochemistry

**Scheme 1:**
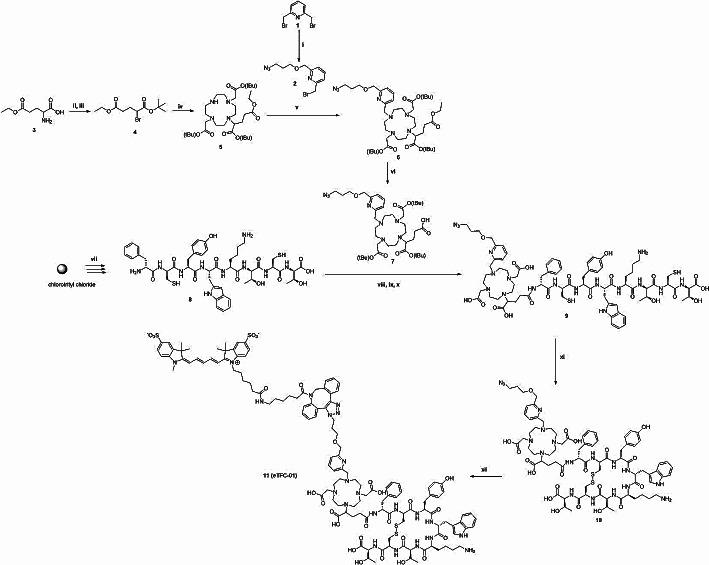
Synthesis of **eTFC-01** (**11**): (i) 3-azidopropanol, potassium *tert*-butoxide, dry THF, overnight, rt, 18%; (ii) Potassium bromide, sodium nitrate, 3 h, 0 °C; (iii) *tert*-Butyl 2,2,2-trichloroacetimidate, dimethylacetamide, boron trifluoride-diethyl ether, overnight, rt, 28%; (iv) DO2A-*tert*-butyl-ester, potassium carbonate, potassium iodide, acetone, overnight, reflux, 49%; (v) **2**, potassium phosphate, potassium iodide, CH_3_CN, reflux, 39%; (vi) NaOH (1 M), 1,4-dioxane, pH 8.5, 3 h, rt, 59%; (vii) SPPS; (viii) **7**, HATU, DIPEA, 16 h, rt; (ix) TFA/H_2_O/TIPS (95:2.5:2.5 v/v/v), 3 h, rt; (x) TFA, 48 h, rt, 6%; (xi) 5% DMSO in 2 mM Gdn.HCl, 100 mM NaH_2_PO_4_, 16 h, rt, 63%; (xii) Sulfo-Cy5 DBCO, H_2_O/can (1:1 v/v), 1 h, rt, 94%

N_3_-Py-DOTAGA-(*t*Bu)_3_ (**7**) was synthesized in five steps (Scheme 1). Starting from 2,6-bis(bromomethyl)pyridine, the azido arm **2** was prepared with a yield of 18% by a nucleophilic substitution of one of the bromides with 3-azidopropanol. The reaction was performed under N_2_ atmosphere to avoid hydrolysis of the remaining bromide (Fig. S1). The following step was the functionalization of L-glutamic acid γ-ethyl ester (**4**) using a Sandmeyer reaction, followed by the *tert*-butylation of the resulting carboxylic acid in presence of *tert*-butyl-trichloroacetimidate (TBTA)(Armstrong et al. 1988). The glutamic arm **4** was obtained with a yield of 28% (Fig. S2). Next, the coupling of **4** to DO2A *tert*-butyl ester yielded mono- and di-substituted products. The major product **5** was isolated by column chromatography with a yield of 49% (Fig S3). Then, the azido arm **2** was conjugated to the macrocyclic intermediate **5**. The reaction was performed in absence of carbonate base to avoid side reaction between the formed CO_2_ and the pyridine ring(Lim et al. 2013). Instead, potassium phosphate was used to give the desired product **6** with a yield of 39% (Fig. S4). The ultimate step was the deprotection of the ethyl group to free a carboxylic acid function allowing the conjugation of **7** to the amine group of the targeting vector. The deprotection was done in basic conditions, however, the pH was maintained between 8 and 9 to avoid concomitant deprotection of the *tert*-butyl groups at higher pH. N_3_-Py-DOTAGA-(*t*Bu)_3_ was obtained with a yield of 59% (Fig. S5).

Synthesis of linear peptide **8** was performed on solid support using a standard Fmoc-based strategy (Scheme 1). The peptide **9** was synthesized by conjugation of N_3_-Py-DOTAGA-(*t*Bu)_3_ to **8** in presence of the coupling agent hexafluorophosphate azabenzotriazole tetramethyl uronium (HATU), followed by the deprotection of the side-chain protecting groups and cleavage from the solid support. An additional deprotection step in neat trifluoroacetic acid (TFA) was necessary to complete the removal of all *t*Bu groups. **9** was obtained with a yield of 6% (Fig. S6) after purification by semi-preparative high-performance liquid chromatography (HPLC). **10** was obtained with a yield of 60% by cyclization of the linear peptide **9** using dimethyl sulfoxide (DMSO)-mediated oxidation in basic conditions (NaH_2_PO_4_) and in presence of guanidine hydrochloride (Fig. S7). **eTFC-01** was obtained with a yield of 94% (Fig. S8) by the conjugation of the near-infrared fluorescence dye (Sulfo-Cy5) to **10** via a strain-promoted azide-alkyne cycloaddition (SPAAC).

To test if the optical proprieties of the NIRF dye were maintained after coupling to the peptide, the absorbance spectrum of **eTFC-01** was compared to the spectrum of the free dye, Sulfo-Cy5 DBCO (Fig. S9). No difference was observed on the spectra of **eTFC-01** and Sulfo-Cy5 DBCO, especially at the excitation and emission wavelengths of 646 nm and 662 nm, respectively. However, a slightly higher signal intensity was noticed for our compound compared to the free dye.

Labeling of **eTFC-01** was performed with [^111^In]InCl_3_ at 90 °C in presence of sodium acetate buffer and a mixture of ascorbic acid and gentisic acid to prevent radiolysis. The radiochemical yield (RCY) and radiochemical purity (RCP) were determined by instant thin layer chromatography (iTLC) and radio-HPLC, respectively (Table 1). [^111^In]In-**eTFC-01** was obtained with a RCY ≥ 99% and RCP ≥ 99% (Fig. S10 and S11).

**Table 1:** RCY, RCP, LogD and stability studies in PBS and mouse serum of [^111^In]In-**eTFC-01**

Compound	RCY (%) (*n* = 3)	RCP (%) (*n* = 3)	LogD_7.4_ (*n* = 3)	PBS (%)^*^			Mouse serum (%)^*^		
				1 h	4 h	24 h	1 h	4 h	24 h
[^111^In]In-**eTFC-01**	99.15 ± 0.04	99.00 ± 0.06	-0.70 ± 0.06	99.0	99.0	99.0	97.9	86.2	71.1
[^68^Ga]Ga-DOTA-TATE			-3.69 ± 0.02^**^						

^*^ Results are expressed as the percentage of intact labeled ligand after incubation at 37 °C (*n* = 1), ^**^ Value from ref (Schottelius et al. 2015).

### In vitro characterization of eTFC-01

**Fig. 1:**
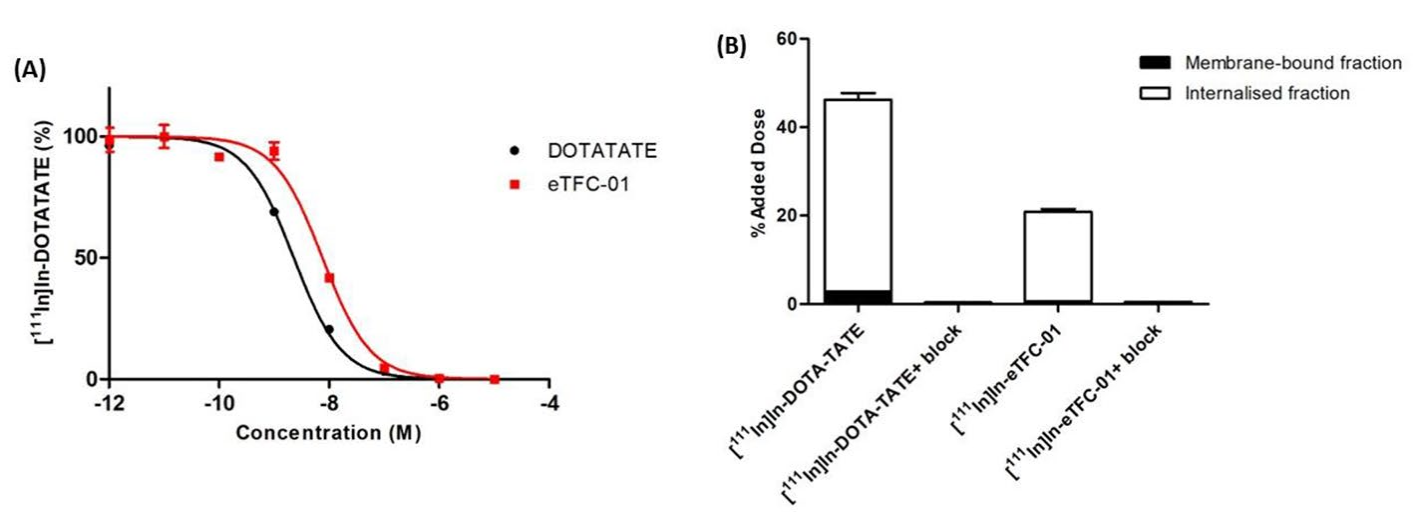
(**A**) IC_50_ curves of **eTFC-01** and DOTA-TATE from a competitive binding assay (*n* = 3). (**B**) Uptake and internalization studies of [^111^In]In-DOTA-TATE and [^111^In]In-**eTFC-01** (*n* = 3). Values are expressed as % of the added dose per 1,000,000 cells (% AD). Uptake of both compounds was blocked with a 50-fold excess of DOTA-TATE

The distribution coefficient, LogD_7.4_, of [^111^In]In-**eTFC-01** was negative, confirming its hydrophilic character (Table 1). No degradation of [^111^In]In-**eTFC-01** was observed when the tracer was incubated in PBS buffer (Fig. S13), however only 71% intact [^111^In]In-**eTFC-01** was found at 24 h in mouse serum (Fig. S12). IC_50_ values were obtained by a competitive binding assay using [^111^In]In-DOTA-TATE, as radioligand, in U2OS cells overexpressing the somatostatin receptor subtype 2 (Fig. 1A). **eTFC-01** exhibited a nanomolar affinity to SSTR2, with an IC_50_ value 2.3-fold higher (*P* < 0.05) than the value of the gold standard DOTA-TATE (8.29 ± 0.51 nM vs. 3.60 ± 0.93 nM, respectively).

Cell uptake studies of [^111^In]In-**eTFC-01** and [^111^In]In-DOTA-TATE were carried out in U2OS.SSTR2 cells (Fig. 1B). The total uptake of [^111^In]In-DOTA-TATE was 2.3-fold higher (*P* < 0.05) than the uptake of [^111^In]In-**eTFC-01** (46.22 ± 4.58% AD vs. 20.87 ± 1.92% AD, respectively), which is in agreement with the IC_50_ values previously reported for those two compounds. We also noticed that the cell uptake was almost completely blocked when an excess of unlabeled DOTA-TATE was added to the incubation medium, demonstrating the specificity of the cell uptake. Most radioactivity was found in the internalized fractions (94% and 97% for [^111^In]In-DOTA-TATE and [^111^In]In-**eTFC-01**, respectively), confirming the agonistic properties of the two compounds.

### In vivo evaluation in H69-tumor bearing mice

**Fig. 2:**
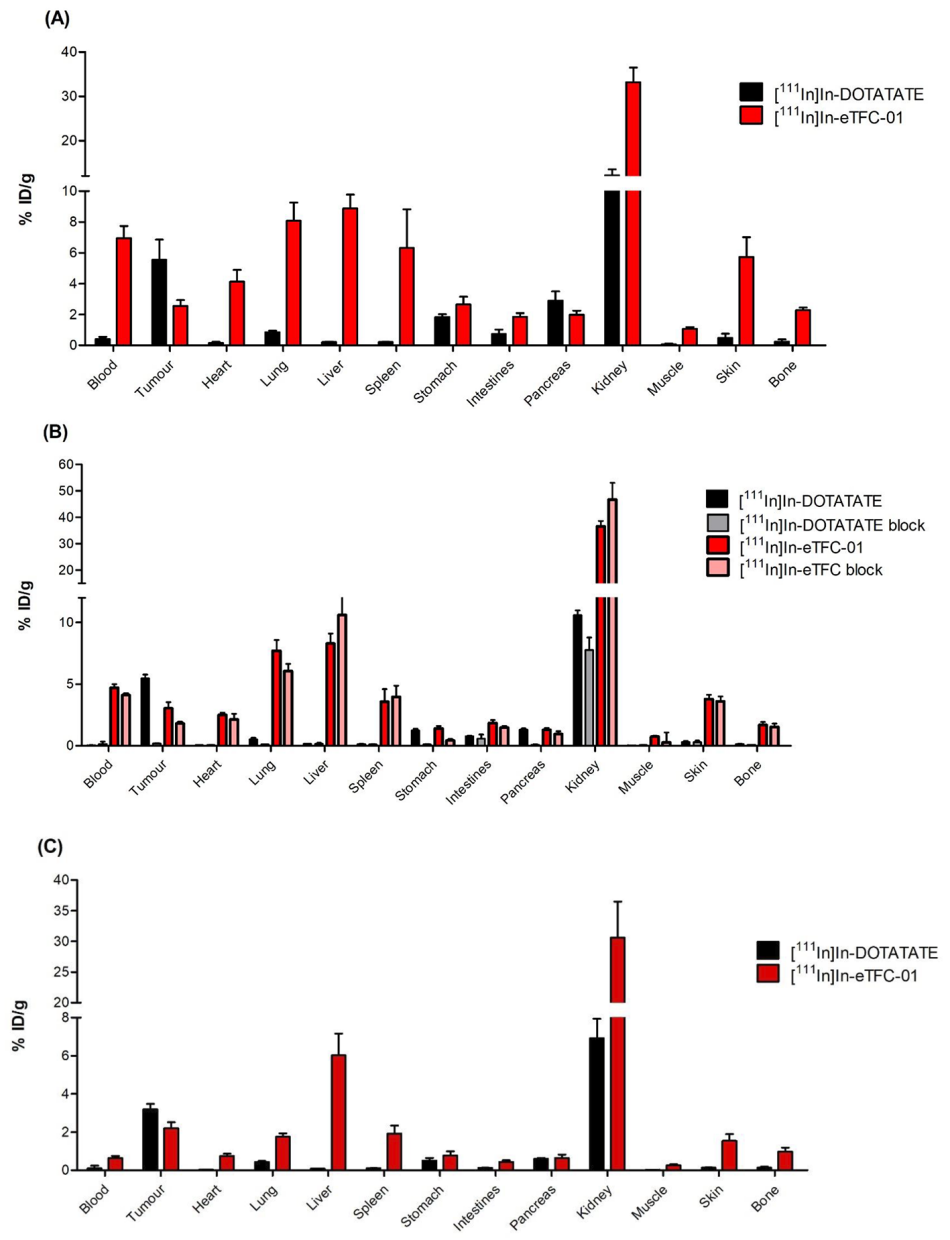
Biodistribution of [^111^In]In-DOTA-TATE and [^111^In]In-**eTFC-01** in H69-xenograft mice at 1 h (**A**), 4 h and 4 h block (**B**), and 24 h (**C**) post-injection (*n* = 4). Uptake in tissues is expressed as the percentage of the injected dose per gram of tissue (% ID/g)

Biodistribution of [^111^In]In-DOTA-TATE and [^111^In]In-**eTFC-01** in H69-xenograft Balb/c nu/nu mice at 3 different time points are depicted in Fig. 2. Uptake was observed in the H69 tumors after administration of [^111^In]In-**eTFC-01**, however this uptake was 54%, 46% and 31% lower at 1 h (*P* < 0.05), 4 h (*P* < 0.05) and 24 h p.i. (*P* < 0.05) than the tumor uptake of [^111^In]In-DOTA-TATE (Table S1 and S2). Co-injection of an excess of DOTA-TATE showed only a tumor uptake reduction of 40% at 4 h p.i. for [^111^In]In-**eTFC-01** compared to the unblocked group (*P* < 0.05), while a reduction of 98% was observed for [^111^In]In-DOTA-TATE (Fig. 2B). Clearance of [^111^In]In-**eTFC-01** through the reticuloendothelial system (liver and spleen) was observed, as well as renal clearance with kidney uptake of [^111^In]In-**eTFC-01** constantly 2.7 to 4.4-fold higher than the renal accumulation of [^111^In]In-DOTA-TATE (*P* < 0.05). [^111^In]In-**eTFC-01** also displayed a long blood half-life compared to [^111^In]In-DOTA-TATE (6.96 ± 0.68% ID/g vs. 0.41 ± 0.12% ID/g at 1 h p.i. and 4.70 ± 0.24% ID/g vs. 0.04 ± 0.01% ID/g at 4 h p.i.). It resulted in high uptake in the highly vascularized non-target organs, such as lungs, skin and heart.

### Ex vivo optical imaging

**Fig. 3:**
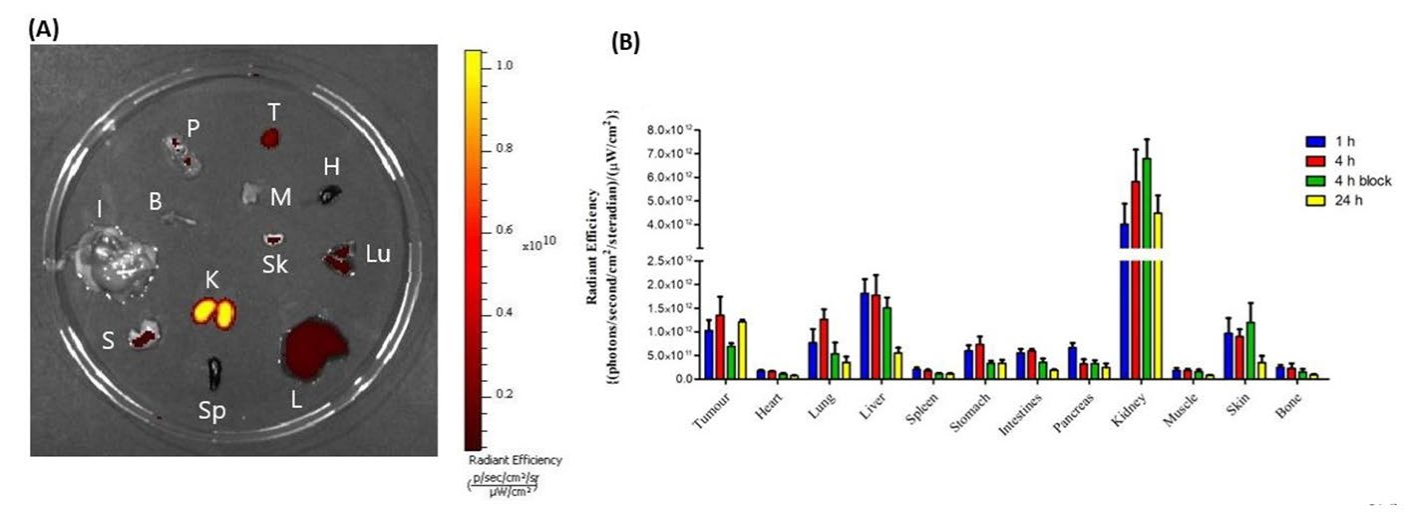
Ex vivo fluorescence imaging of a subset of organs using the IVIS imaging system after dissection of H69-xenografted Balb/c nu/nu mice previously injected with [^111^In]In-**eTFC-01**. (**A**) Representative example of an image of a petri dish containing the various organs. Organ list: tumor (T), heart (H), lung (Lu), liver (L), spleen (Sp), stomach (S), kidneys (K), skin (Sk), intestine (I), bone (B), pancreas (P) and muscle (M). (**B**) Quantification of the fluorescent signal intensities in radiant efficiency {(p/sec/cm^2^/sr)/(μW/cm^2^)}

Following the ex vivo biodistribution, a subset of organs and the tumor were imaged to confirm the localization of the dye (Fig. 3 and Table S3). Overall, the results indicated that the fluorescence intensities of the organs generally followed the same trend as the radioactivity uptake.

## Discussion

Surgery plays a crucial role in improving the survival of patients with neuroendocrine tumors, even in cases where metastasis has occurred. Precision in localizing the primary tumor and metastatic lesions is essential, prompting the development of innovative imaging techniques. The combination of radioactive and optical imaging offers a promising approach, enabling the localization of tumors with the radioactive images (SPECT or PET) followed by precise removal using fluorescence-guided imaging. However, designing a dual-modality tracer with comparable or closely matching pharmacokinetic profile to mono-modality tracers is a challenge. Typically, introduction of a lysine to the *N*-terminal of a peptide vector for coupling both the chelate and the dye can lead to loss of affinity to the target(Handula et al. 2022a, b). To circumvent this challenge, we designed a dual-labeled tracer using the chelate as linker between the dye and the peptide. This approach has previously demonstrated minimal or no impact on binding affinity compared to the parent peptide, providing an effective solution to maintain target affinity The synthesis of our trifunctional chelate is detailed in Scheme 1. Briefly, we initiated the synthesis from the DO2A-*tert*-butyl ester, which has 2 free positions available for derivatization. The two free positions being distant to each other minimized steric hindrances. One building block, termed the Glu arm (**4**), was used to attach the peptide during the solid-phase peptide synthesis (SPPS), similarly to other chelates. The second building block, the pyridine arm (**2**), was used for the attachment of the fluorescence dye and could also contributed to the metal complexation, therefore enhancing the stability of the labeled compound(Chen et al. 2019; Wynn and Hegedus 2000). The synthesis of the pyridine arm **2** started with the mono-substitution of the 2,6-bis(bromomethyl)pyridine with 3-azidopropanol under basic conditions and in nitrogen atmosphere to avoid bromide hydrolysis. In this reaction, traditional inorganic bases like K_2_CO_3_ or Cs_2_CO_3_ were unsuitable due to the formation of a pyridine-CO_2_ complex (+ 44 in mass spectrometry) For the same reason, *N, N*-diisopropylethylamine (DIPEA) was used for the coupling of the pyridine arm **2** to compound **5**. The synthesis of the Glu arm **4** and the chelate **5** followed the procedure described by Ghosh et al. (Ghosh et al. 2017a, b). The final deprotection of the ethyl group presented challenges, with pH control proving crucial. Maintaining the pH between 8 and 9 was essential to achieve successful deprotection, preventing *tert*-butyl deprotection at higher pH or inhibiting deprotection at lower pH.

Next, the synthesis of the SSTR2-targeted dual-labeled tracer was initiated according to the procedure for the preparation of compounds like DOTA-TATE, involving the following steps: Fmoc-based SPPS of the linear peptide, cyclization, coupling of the chelate, and final cleavage/global deprotection. We initially attempted to follow this sequence, however, the coupling of the chelate was inefficient, probably due to the steric hindrance between the modified chelate and the cyclic peptide. Therefore, our approach required a modification in the order of these steps. We adjusted the synthesis steps as follows: Fmoc-based SPPS of the linear peptide, coupling of the chelate, cyclization and cleavage/global deprotection. However, uncomplete deprotection of the *tert*-butyl group of the chelate was noticed, even when harsh acidic conditions (e.g., neat TFA or HCl) were used. Consequently, **eTFC-01** was prepared via the following synthetic route: Fmoc-based SPPS of the linear peptide, coupling of the chelate, cleavage/global deprotection and cyclization. To achieve successful cyclization, various conditions were explored, including attempts with Tl(TFA)_3_ or I_2_ to form the disulfide bridge. Unfortunately, under these conditions, side reactions occurred, such as chelate complexation with thallium(Hijnen et al. 2011) or the interaction of the iodine with pyridine to form a stable molecular Pyr*I_2_ complex(Tassaing and Besnard 1997). Therefore, new reaction conditions were investigated, specifically using oxidation with DMSO, as previously described by Góngora-Benítez et al. This alternative approach proved effective, resulting in the formation of the cyclic peptide **10** without the occurrence of undesirable side-products (Góngora-Benítez et al. 2011). Finally, **eTFC-01** was obtained after introduction of Sulfo-Cy5 DBCO to the azido-peptide **10** via copper-free click chemistry. Considering all functional groups present in the peptide-chelator conjugate **10**, we decided to selectively couple the dye on the chelator at the late stage of the synthesis via click chemistry. We selected the strain promoted alkyne-azide cycloaddition (SPAAC), instead of the classical copper-catalyzed azide-alkyne Huisgen cycloaddition, to avoid the use of copper and its potential complexation by the chelate(Chen et al. 2012; Cai and Anderson 2014; Bauer et al. 2023).

The radiolabeling process of **eTFC-01** with indium-111 was efficiently carried out at 90 °C, maintaining a pH between 5 and 6, resulting in a high radiochemical yield and purity. These radiolabeling conditions are particularly well-suited for labeling peptides (Richter and Wuest 2014). Importantly, the presence of quenchers (e.g., ascorbic acid and gentisic acid) in the formulation prevented the radiolytic degradation of [^111^In]In-**eTFC-01**(Larenkov et al. 2023). The stability studies revealed that [^111^In]In-**eTFC-01** exhibited no signs of degradation in phosphate-buffered saline and a good stability in mouse serum at 24 h. This demonstrated the inertness of the compound towards radiolysis and relative resistance to peptidase digestion, confirming its stability under physiological conditions. [^111^In]In-**eTFC-01** showed a significant decreased of hydrophilicity compared to the parent [^68^Ga]Ga-DOTA-TATE (LogD_7.4_ = -3.69 ± 0.02)(Schottelius et al. 2015). This decrease in hydrophilicity can be attributed to the modification of the chelate, particularly the pyridine arm. The introduction of the DBCO group, known to be lipophilic, may explain the increase of the LogD_7.4_ value(Kettenbach et al. 2018). The dual-labeled tracer **eTFC-01** demonstrated a binding affinity to SSTR2 that was two-fold lower than the gold standard DOTA-TATE. Remarkably, the incorporation of the dye via the chelate, positioned away from the binding site, did not result in an important loss of the binding to SSTR2. Furthermore, its affinity to SSTR2 is significantly better than the affinity previously reported for the dual-labeled SSTR2 ligands using a lysine residue as anchor point to introduce the dye and the chelator. These ligands typically exhibited binding affinities 25 to 100-fold worse than the affinity of the parent peptides(Kuil et al. 2010), as observed by Ghosh et al. (EC_50_ = 455 ± 299 vs. 11.0 ± 0.8 for Cu-DA(IR800)-TOC vs. Ga-DOTATOC) or Santini et al. (IC_50_ = 487.7 vs. 20.35 for Cy_5_-DTPA-Tyr^3^-octreotate vs. Tyr^3^-octreotate), indicating that this approach induces more disturbances to the binding [^111^In]In-**eTFC-01** displayed a total cell uptake two-fold lower than [^111^In]In-DOTA-TATE, consistent with the results of the binding studies. Internalization studies confirmed the agonistic properties of the ligand, aligning with the expectations for an octreotate derivative. The SSTR2 specificity of [^111^In]In-**eTFC-01** was confirmed by the absence of uptake following blocking with an excess of DOTA-TATE.

In the evaluation conducted on a human small-cell lung cancer (NCI-H69) xenograft model overexpressing SSTR2, [^111^In]In-**eTFC-01** and [^111^In]In-DOTA-TATE were compared. A higher amount of peptide for the dual-labeled tracer was injected compared to DOTA-TATE (2 nmol vs. 0.5 nmol, respectively). This amount of **eTFC-01** is corresponding to the level of tracer typically administered for preclinical optical imaging to warrant good fluorescent signal from the tumor and tumor-to-background contrast (Vargas et al. 2019; Edwards et al. 2008). We decided to inject a different mass of DOTA-TATE, since previous studies on dose optimization demonstrated that the tumor uptake of DOTA-TATE is optimal with a lower amount of peptide(Jong et al. 1999). Tumor uptake of [^111^In]In-**eTFC-01** remained consistently around 2–3% ID/g from 1 h to 24 h post injection, while tumor uptake of [^111^In]In-DOTA-TATE decreased from ∼ 5% ID/g at 1 h to ∼ 3% ID/g at 24 h. Notably, in a study by Ghosh et al., their dual-labeled tracer, [^64^Cu]Cu-MMC(IR800)-TOC, exhibited a tumor uptake of 5% ID/g at 4 h post injection in AR42J tumors. However, when comparing the tumor uptake of [^111^In]In-**eTFC-01** and [^64^Cu]Cu-MMC(IR800)-TOC, it must be taken into account that the AR42J cell line is known to have a 3-fold higher SSTR2 expression than the NCI-H69 cell line(Taylor et al. 1994). Furthermore, [^64^Cu]Cu-MMC(IR800)-TOC demonstrated a slightly better stability in mouse serum (98% for [^64^Cu]Cu-MMC(IR800)-TOC and 86% for [^111^In]In-**eTFC-01** at 4 h). This difference could eventually be explained by a higher radiosensibility of our fluorescence dye, Sulfo-Cy5, compared to IRDye800CW(Hernandez et al. 2017). A prolonged blood circulation was observed for [^111^In]In-**eTFC-01** at 1 and 4 h p.i. compared to [^111^In]In-DOTA-TATE. It could originate from the decrease of hydrophilicity of the ligand, facilitating interaction with albumin, a characteristic accentuated by the presence of the cyanine dye known for its non-covalent interactions with albumin(Kuil et al. 2011; Kashin and Tatikolov 2009). As a result, the background signal in vascularized tissues (e.g., heart, lung, skin) was high at 1 and 4 h p.i., and decreased at 24 h p.i. due to the clearance of [^111^In]In-**eTFC-01** from the blood. A blocking study was performed at 4 h p.i. and showed a reduction of only 40% of the tumor uptake. This result was not in concordance with the in vitro blocking studies (95% drop of the cell uptake in presence of an excess of DOTA-TATE). It might be due to the high vascularization of the tumors and long residence time of the tracer in the blood(Ohshika et al. 2021). Renal clearance was identified as the primary excretion route of our tracer, with a 3-fold higher uptake in the kidneys compared to [^111^In]In-DOTA-TATE, possibly attributed to the additional charge introduced by the cyanine, as observed by Santini et al. In addition to renal clearance, excretion via the reticuloendothelial system (liver and spleen) was observed for our tracer. The change in hydrophilicity of **eTFC-01** may contribute to this observation, as studies have shown that targeting vectors tend to be cleared more through the reticuloendothelial system than the renal system when the LogD value is increasing(Tafreshi et al. 2021). Remarkably, a similar distribution was noted for the fluorescence signal of [^111^In]In-**eTFC-01**, emphasizing the versatility of the ligand for both radioactive imaging (SPECT or PET) and fluorescence-guided surgery.

We considered that the low tumor uptake combined with the long blood circulation and excretion profile would have likely resulted in a poor tumor visualization in imaging studies. Therefore, for ethical reasons, we decided no to perform additional SPECT and optical imaging studies with **eTFC-01**[76]. While the in vitro results were encouraging, improvement of the biodistribution profile can be achieved through chemical modifications of the dual-labeled tracer. First, the DBCO function could be substituted by a standard alkyne group, thereby using a copper-catalyzed azide–alkyne cycloaddition (CuAAC) instead of SPAAC. To prevent complexation of the copper, it can be removed from the reaction by treatment with sodium sulfide upon completion of the click reaction. Thus, the end-product would not contain the two aromatic rings of DBCO, which would result in an enhanced hydrophilicity and consequently a more favorable excretion pathway(Kettenbach et al. 2018). Alternatively, the charge of the fluorescent dye could be changed. The current dye carries a negative charge, influencing the net charge of **eTFC-01.** Conjugation of a zwitterionic dye could influence the uptake in background tissues by maintaining a neutral charge. Studies using zwitterionic dyes, as opposed to conventional charged dyes, demonstrated improvements in the background signal and excretion of the fluorescent tracers(Choi et al. 2011, 2013; Hernandez Vargas et al. 2022). Once the structural modification of the tracer will be completed, nuclear and fluorescence imaging will be performed, as well as fluorescence-guided tumor resection, to demonstrate the potential of our synthetic approach to generate dual-labeled tracers with optimal properties. The amount of tracer will also be studied to determine how the pharmacokinetics and tissue distribution is affected by the mass of peptide injected.

## Conclusions

We reported a new design approach for the synthesis of multimodal imaging tracers based on a trifunctional chelate serving as a functional linking unit. The synthesis of our trifunctional chelate and chelate-bridged hybrid tracer was successful after optimization of the synthetic route. **eTFC-01**, showed good in vitro SSTR2-affinity and uptake/internalization compared to DOTA-TATE, demonstrating the potential of our chelate as linker. Our dual-labeled tracer exhibited promising capabilities in targeting SSTR2 tumors in vivo, facilitating both nuclear and fluorescence detection modalities. However, challenges arise from the significant uptake in nontarget organs observed in biodistribution studies, particularly attributable to prolonged blood circulation and liver excretion pathways. This elevated background diminishes the efficacy of **eTFC-01**, particularly in the context of fluorescence-guided surgery, where precise detection of tumor margin and lymph node metastases is critical. To address these limitations, it is imperative to undertake a thorough optimization of the molecular structure of **eTFC-01**. This optimization aims to enhance the tumor-to-background signal ratio, thereby improving imaging contrast. Once the optimization process is successfully completed, the revised dual-labeled tracer will be evaluated for its accuracy in the visualization of NET tumors by SPECT/PET and fluorescence imaging. Moreover, it will enable proof-of-concept studies of fluorescence-guided surgery in mice, offering more precise and effective tumor resections. Furthermore, the versatility of the trifunctional chelate strategy employed in our design can be extended beyond SSTR2-targeted imaging.

### Electronic supplementary material

Below is the link to the electronic supplementary material.

Supplementary Material 1: The following supporting information can be downloaded at: Synthesis and characterization of compound **2–11**; ^1^H NMR, ^13^C NMR and ESI-MS spectra of **2**, **4**, **5**, **6** and **7**; LC chromatograms and ESI-MS spectra of **9**, **10** and **11**; Absorbance spectrum of **eTFC-01** and sulfo-Cyanine 5 DBCO at two concentrations; iTLC spectrum and radio-HPLC chromatogram of [^111^In]In-**eTFC-01**. PBS and mouse serum stability studies of [^111^In]In-**eTFC-01.** Ex vivo biodistribution data of [^111^In]In-DOTA-TATE at 1, 4, 4 h block and 24 h post-injection (*n* = 4 mice/group); Ex vivo biodistribution data of [^111^In]In-**eTFC-01** at 1, 4, 4 h block and 24 h post-injection (*n* = 4 mice/group); Ex vivo fluorescence measurements of [^111^In]In-**eTFC-01** at 1, 4, 4 h block and 24 h post-injection (*n* = 4 mice/group).

## Data Availability

All data generated and analyzed during this study are included in this published article. Supporting information is provided containing additional data. Additional information is available from the corresponding author upon reasonable request.
