# Supplementary material for: eTFC-01: a dual-labeled chelate-bridged tracer for SSTR2-positive tumors
Source: EJNMMI Radiopharm Chem. 2024 May 22;9:44. doi: 10.1186/s41181-024-00272-0 (PMC11111636; doi:10.1186/s41181-024-00272-0)
Supplement: Supplementary file 1 — Supplementary Material 1: The following supporting information can be downloaded at: Synthesis and characterization of compound 2–11; 1H NMR, 13C NMR and ESI-MS spectra of 2, 4, 5, 6 and 7; LC chromatograms and ESI-MS spectra of 9, 10 and 11; Absorbance spectrum of eTFC-01 and sulfo-Cyanine 5 DBCO at two concentrations; iTLC spectrum and radio-HPLC chromatogram of [111In]In-eTFC-01. PBS and mouse serum stability studies of [111In]In-eTFC-01. Ex vivo biodistribution data of [111In]In-DOTA-TATE at 1, 4, 4 h block and 24 h post-injection (n = 4 mice/group); Ex vivo biodistribution data of [111In]In-eTFC-01 at 1, 4, 4 h block and 24 h post-injection (n = 4 mice/group); Ex vivo fluorescence measurements of [111In]In-eTFC-01 at 1, 4, 4 h block and 24 h post-injection (n = 4 mice/group). [file 41181_2024_272_MOESM1_ESM.docx]

eTFC-01: a Dual-Labeled Chelate-Bridged Tracer for SSTR2-Positive Tumors

**Dylan Chapeau^1,2^, Savanne Beekman^1,2^, Maryana Handula^1,2^, Erika Murce ^1,2^, Corrina de Ridder^1,2^, Debra Stuurman^1,2^, Yann Seimbille^1,2,3,*^**

1. Erasmus MC, University Medical Center Rotterdam, Department of Radiology and Nuclear Medicine, Rotterdam, The Netherlands
2. Erasmus MC Cancer Institute, Rotterdam, The Netherlands
3. TRIUMF, Life Sciences Division, Vancouver, Canada

***** Correspondence: y.seimbille@erasmusmc.nl ; Tel: +31 10-703-8961

Figure S1: ^1^H NMR, ^13^C NMR and ESI-MS spectra of 2-((3-azidopropoxy)methyl)-6-(bromomethyl)pyridine (**2**). 3

Figure S2: ^1^H NMR and ^13^C NMR spectra of 5-benzyl-1-*tert*-butyl 2-bromopentanedioate (**4**). 4

Figure S3: ^1^H NMR, ^13^C NMR and ESI-MS spectra of 1-(*tert*-butyl) 5-ethyl 2-(4,10-bis(2-(*tert*-butoxy)-2-oxoethyl)-1,4,7,10-tetraazacyclododecan-1-yl)pentanedioate (**5**). 5

Figure S4: ^1^H NMR, ^13^C NMR and ESI-MS spectra of 1-(*tert*-butyl) 5-ethyl 2-(7-((6-((3-azidopropoxy)methyl)pyridine-2-yl)methyl)-4,10-bis(2-(*tert*-butoxy)-2-oxoethyl)-1,4,7,10-tetraazacyclododecan-1-yl)pentanedioate (**6**). 7

Figure S5**:** ^1^H NMR, ^13^C NMR and ESI-MS spectrum of N_3_-Py-DOTAGA-(*t*Bu)_3_ (**7**) 8

Figure S6: LC chromatogram and ESI-MS spectrum of N_3_-Py-DOTAGA-D-Phe-Cys-Tyr-D-Trp-Lys-Thr-Cys-Thr-OH (**9**). 9

Figure S7: LC chromatogram and ESI-MS spectrum of N_3_-Py-DOTAGA-D-Phe-cyclo[Cys-Tyr-D-Trp-Lys-Thr-Cys]-Thr-OH (**10**). 9

Figure S8: LC chromatogram and ESI-MS spectrum of **eTFC-01** (**11**). 10

Figure S9: Absorbance spectrum of **eTFC-01** and sulfo-Cyanine 5 DBCO at two concentrations: 10^-4^ M (G5 and G9 for **eTFC-01** and sulfo-Cyanine 5 DBCO, respectively) and 10^-5^ M (G3 and G7 for **eTFC-01** and sulfo-Cyanine5 DBCO, respectively) 11

Figure S10: iTLC spectrum of [^111^In]In-**eTFC-01**. 11

Figure S11: Radio-HPLC chromatogram of [^111^In]In-**eTFC-01***.* 12

Figure S12: Stability of [^111^In]In-**eTFC-01** in mouse serum 12

Figure S13: Stability of [^111^In]In-**eTFC-01** in PBS 13

Table S1: Ex vivo biodistribution data of [^111^In]In-DOTA-TATE at 1, 4, 4 h-block and 24 h post-injection (n = 4 mice/group). Data are represented as percentage of injected dose per gram of tissue (% ID/g). 18

Table S2: Ex vivo biodistribution data of [^111^In]In-**eTFC-01** at 1, 4, 4 h-block and 24 h post-injection (n = 4 mice/group). Data are represented as percentage of injected dose per gram of tissue (% ID/g). 19

Table S3: Ex vivo fluorescence measurements of [^111^In]In-**eTFC-01** at 1, 4, 4 h-block and 24 h post-injection (n = 4 mice/group). Data are represented as average radiant efficiency [10^10^ x (photons/second/cm^2^/steradian)/(mW/cm^2^)] 19


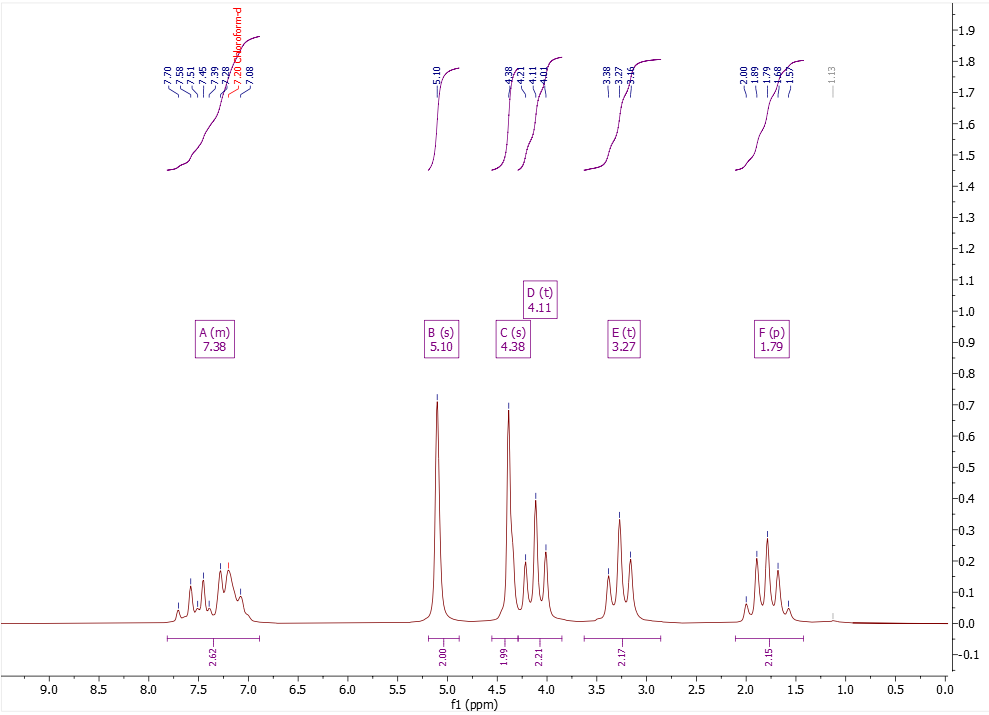


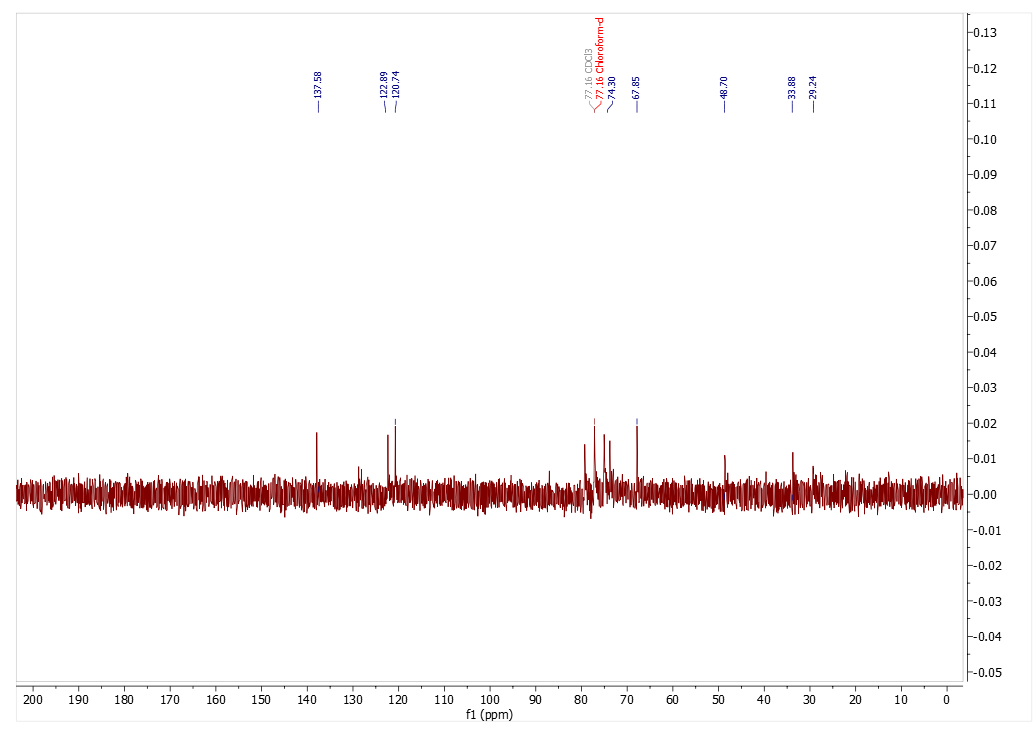


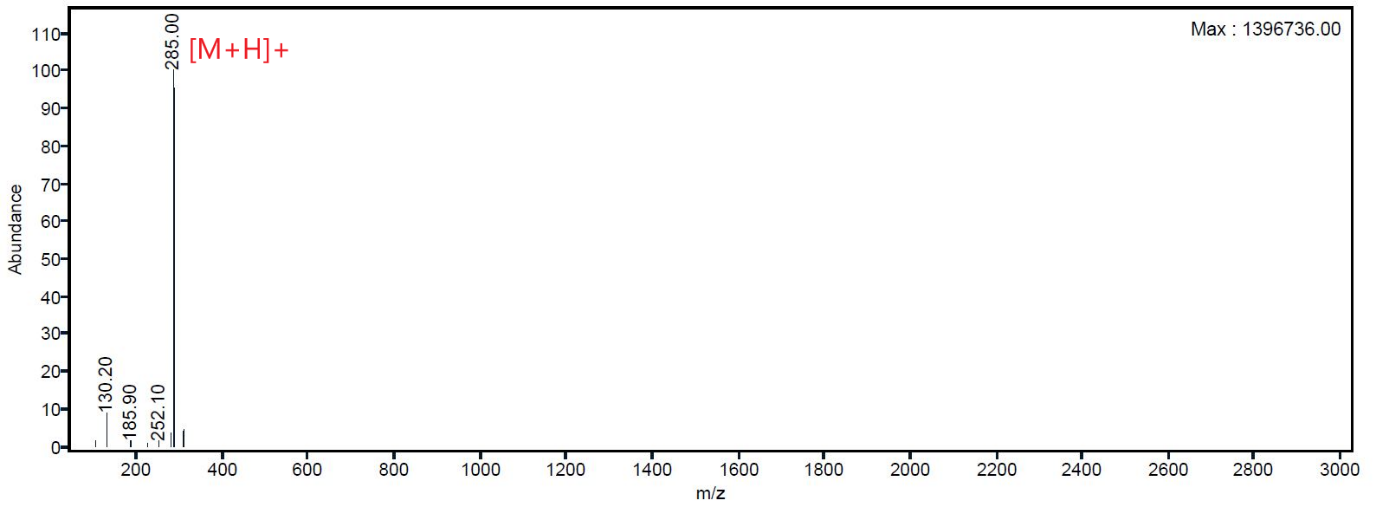


Figure S1: ^1^H NMR, ^13^C NMR and ESI-MS spectra of 2-((3-azidopropoxy)methyl)-6-(bromomethyl)pyridine (**2**).


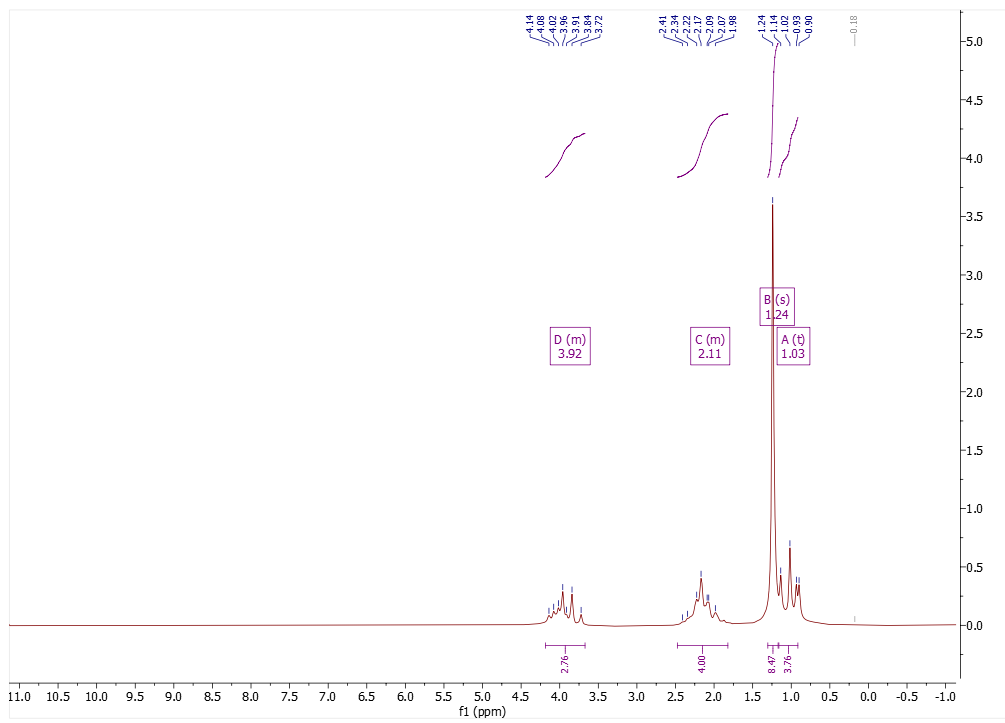


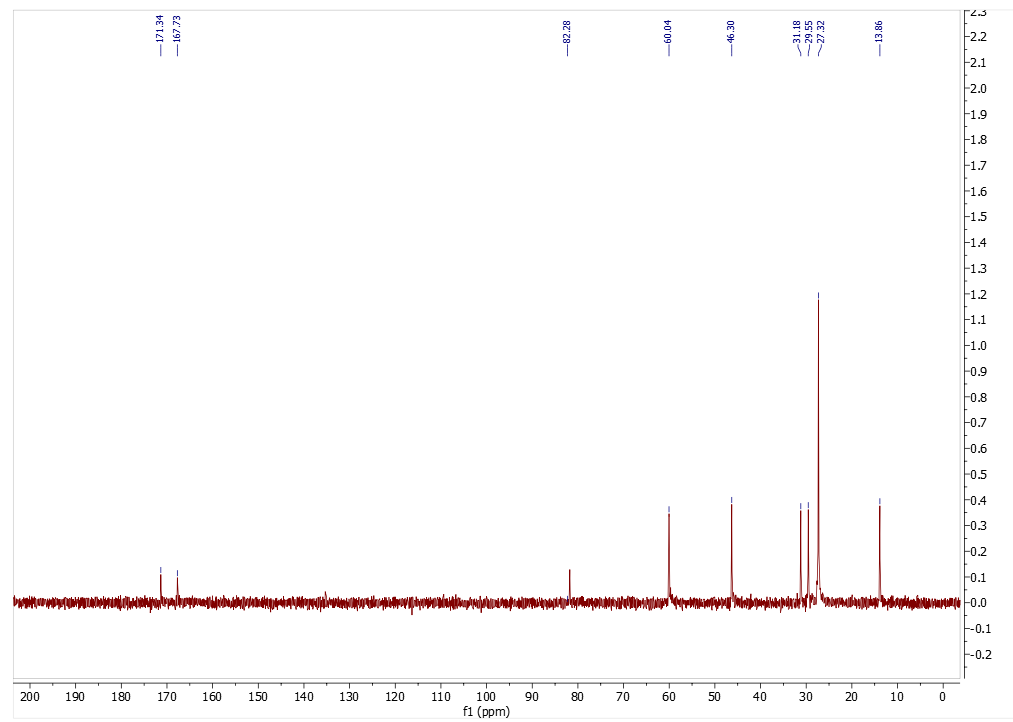


Figure S2: ^1^H NMR and ^13^C NMR spectra of 5-benzyl-1-*tert*-butyl 2-bromopentanedioate (**4**).


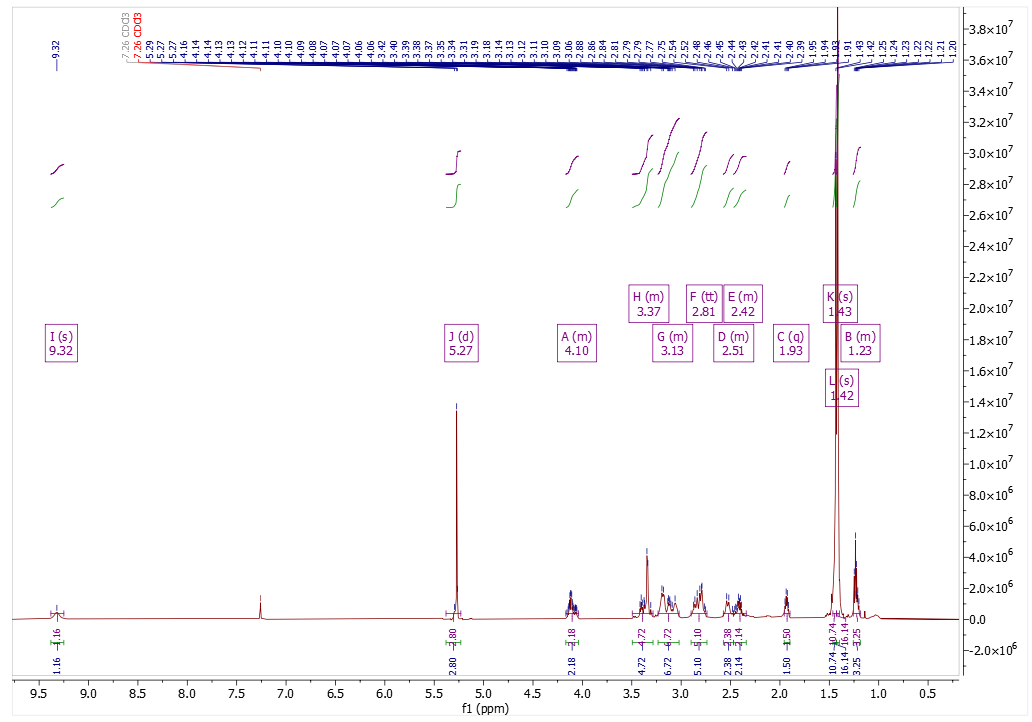


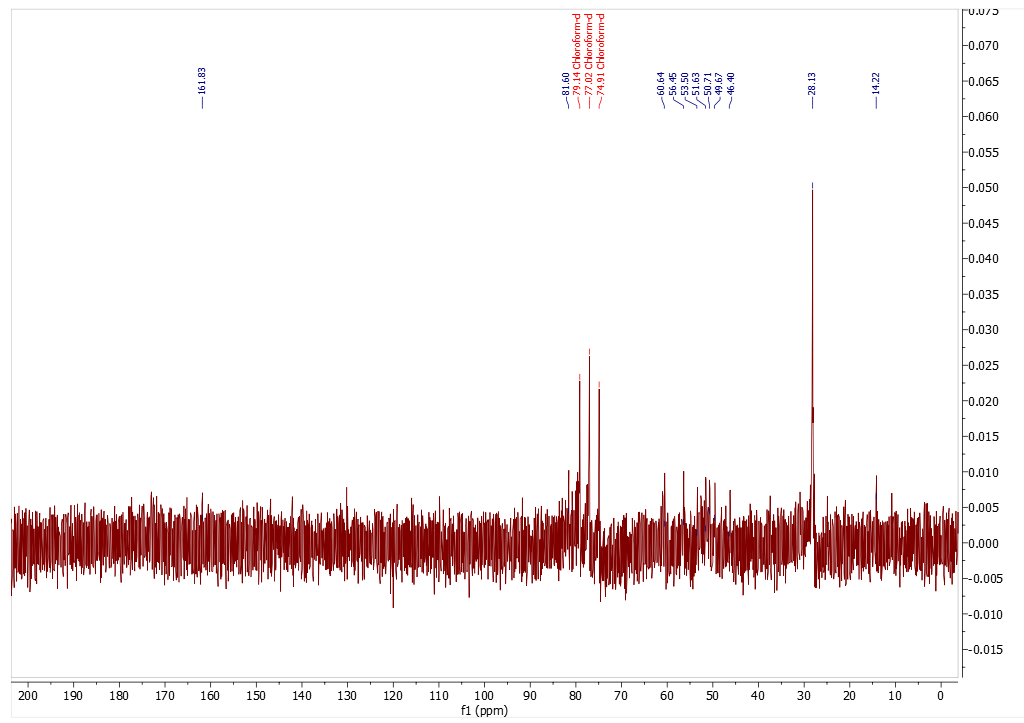


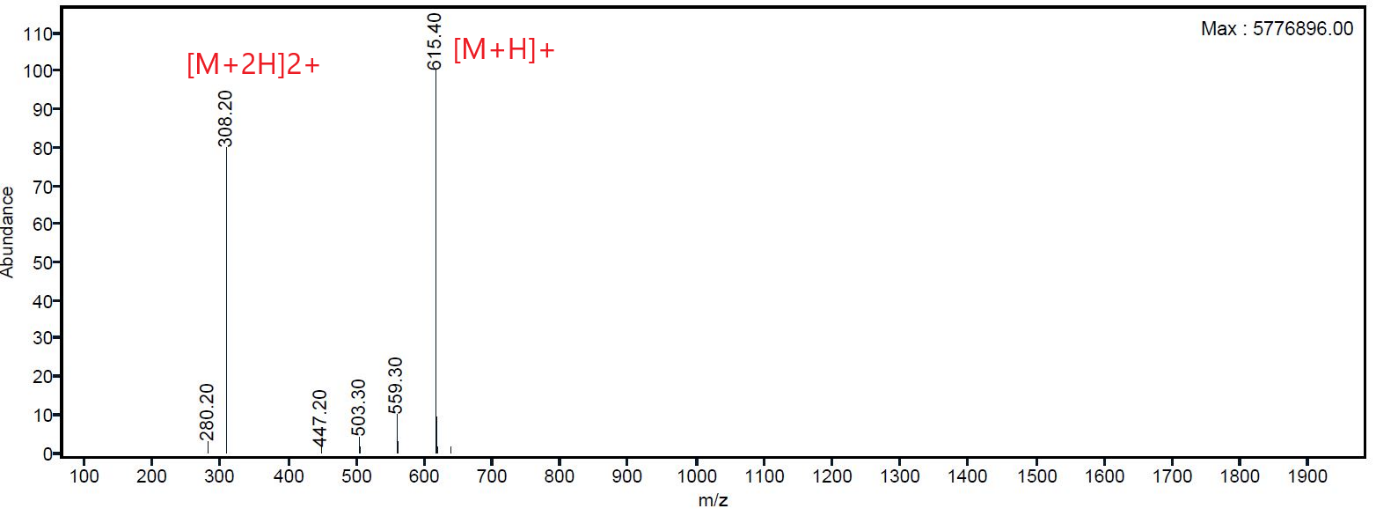


Figure S3: ^1^H NMR, ^13^C NMR and ESI-MS spectra of 1-(*tert*-butyl) 5-ethyl 2-(4,10-bis(2-(*tert*-butoxy)-2-oxoethyl)-1,4,7,10-tetraazacyclododecan-1-yl)pentanedioate (**5**).


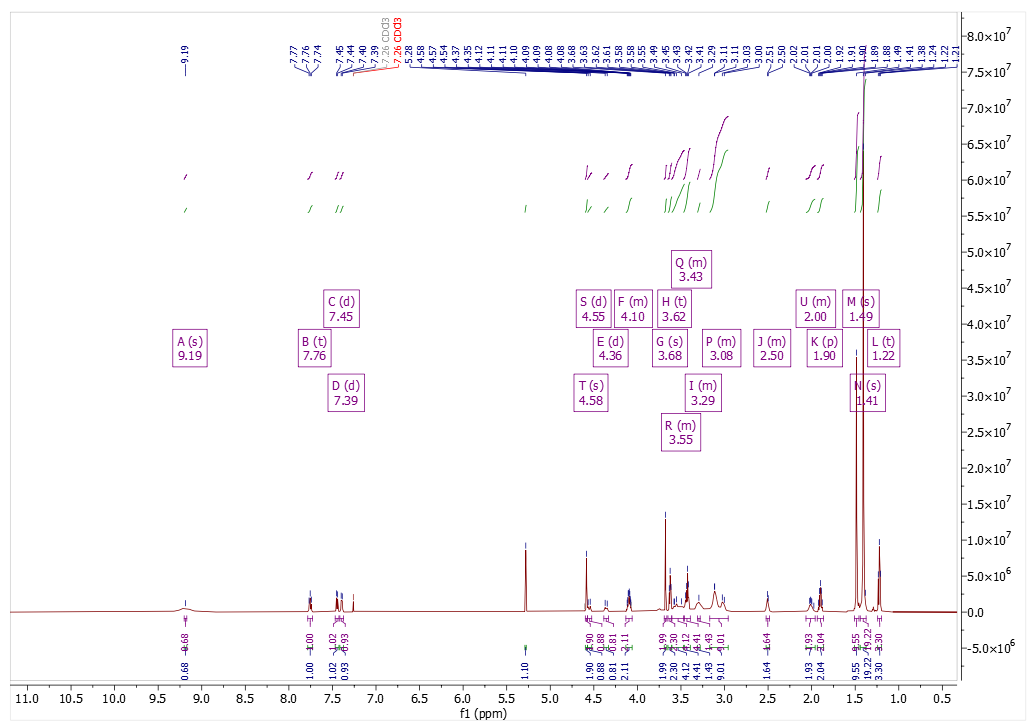


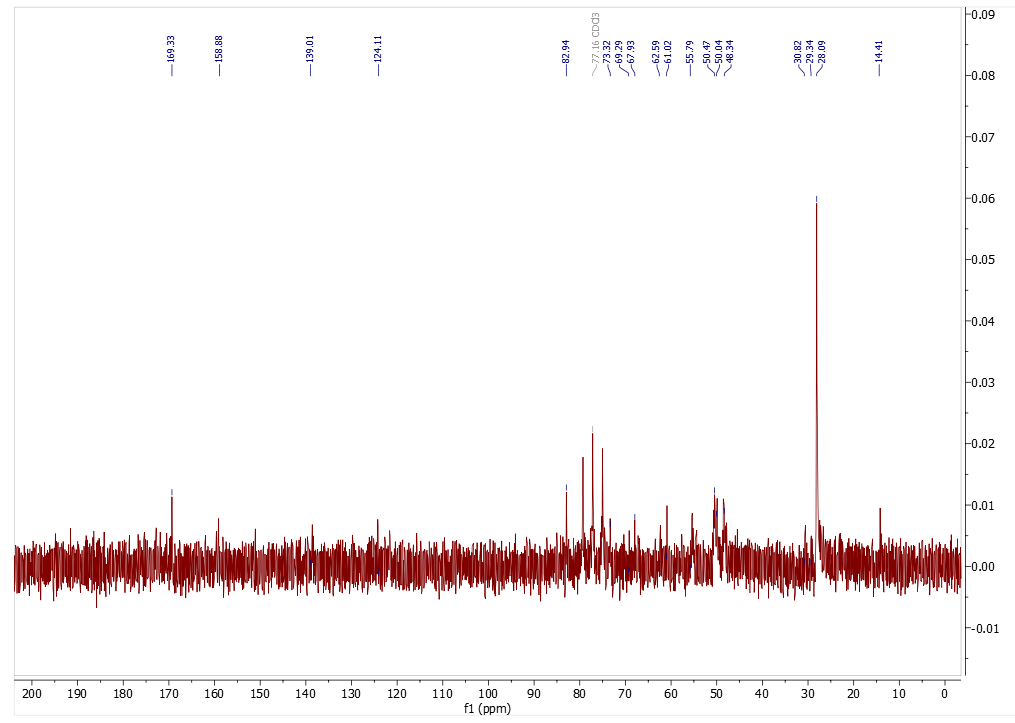


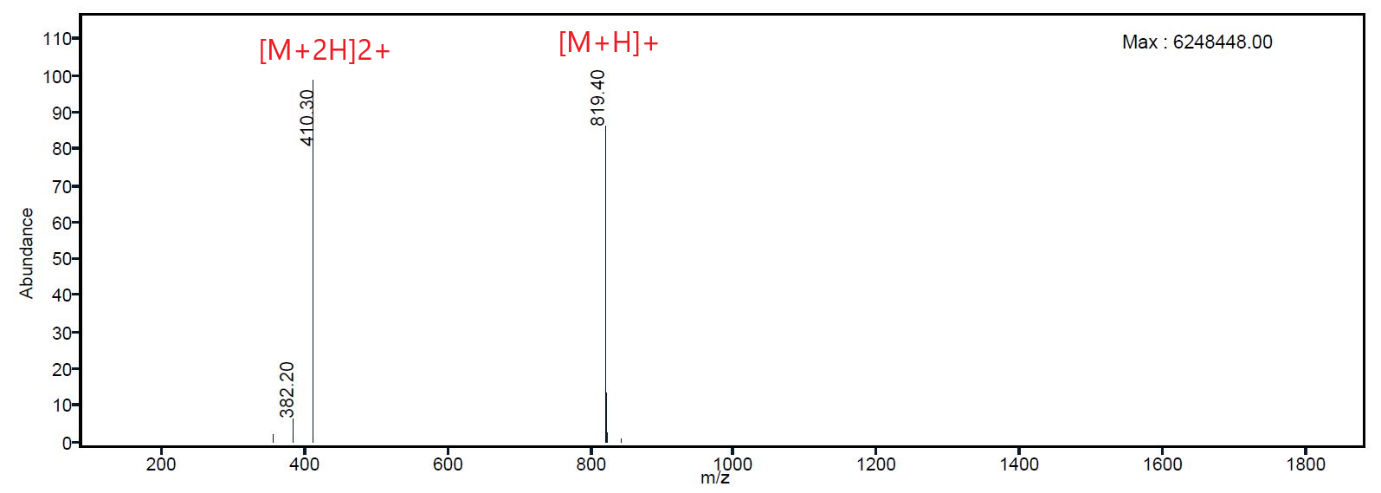


Figure S4: ^1^H NMR, ^13^C NMR and ESI-MS spectra of 1-(*tert*-butyl) 5-ethyl 2-(7-((6-((3-azidopropoxy)methyl)pyridine-2-yl)methyl)-4,10-bis(2-(*tert*-butoxy)-2-oxoethyl)-1,4,7,10-tetraazacyclododecan-1-yl)pentanedioate (**6**).


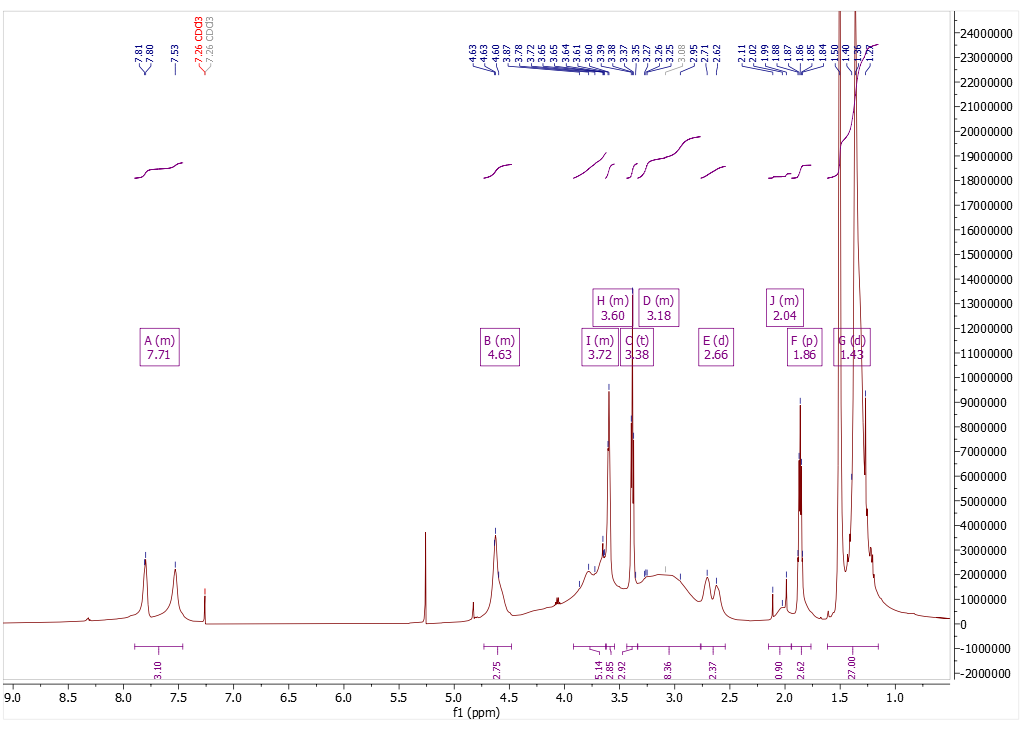


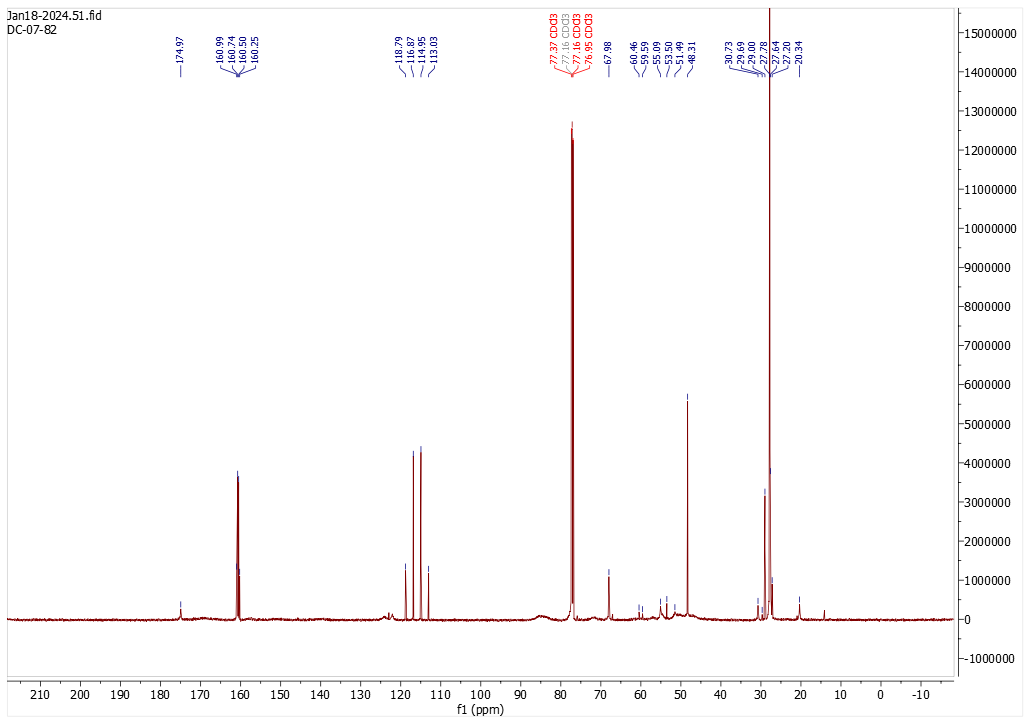


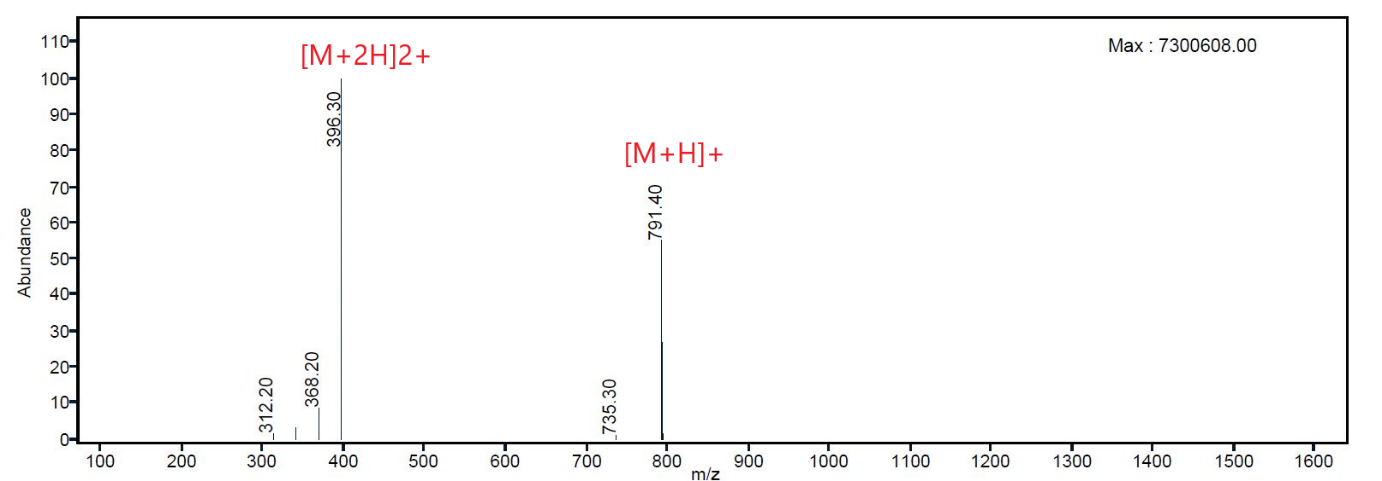


Figure S5**:** ^1^H NMR, ^13^C NMR and ESI-MS spectrum of N_3_-Py-DOTAGA-(*t*Bu)_3_ (**7**)


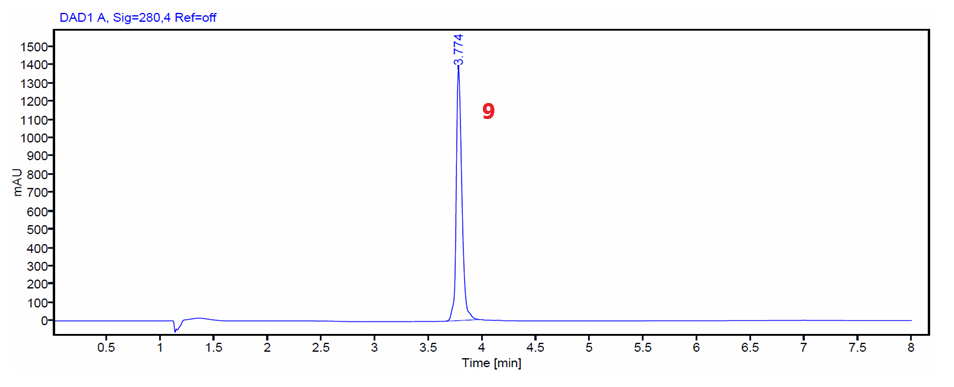


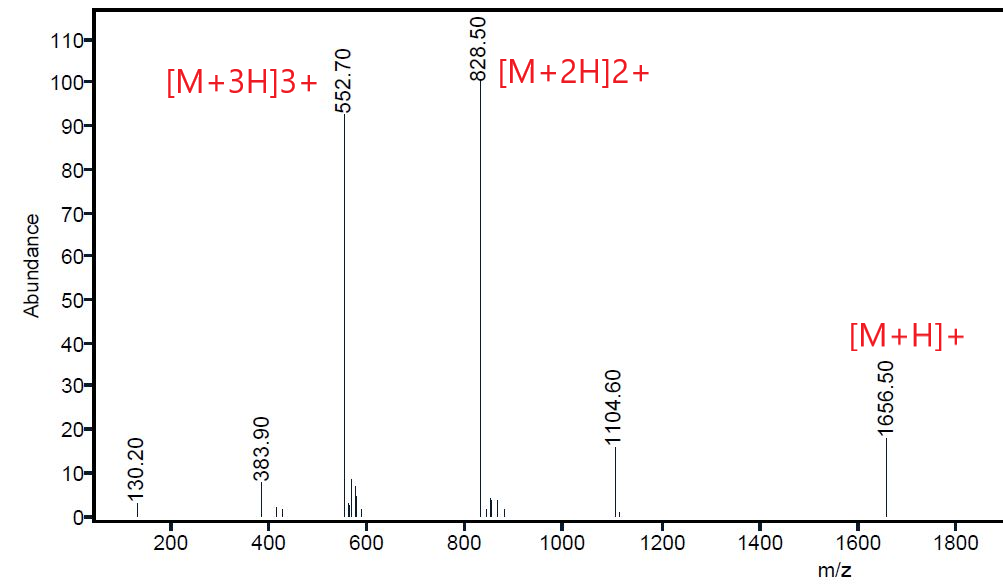


Figure S6: LC chromatogram and ESI-MS spectrum of N_3_-Py-DOTAGA-D-Phe-Cys-Tyr-D-Trp-Lys-Thr-Cys-Thr-OH (**9**).


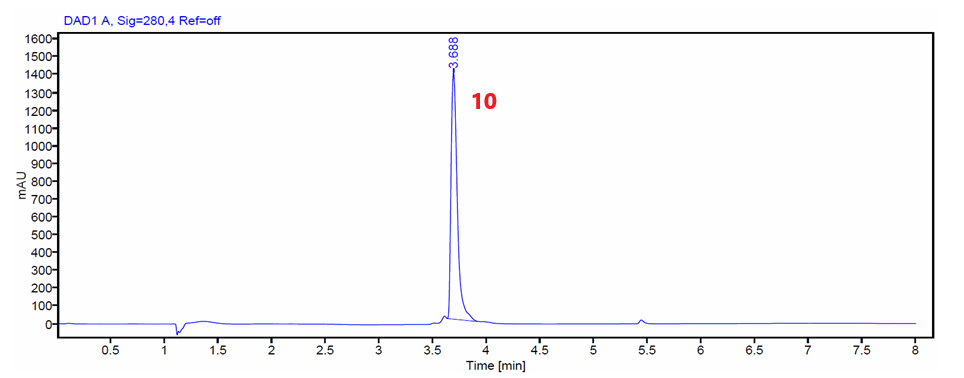


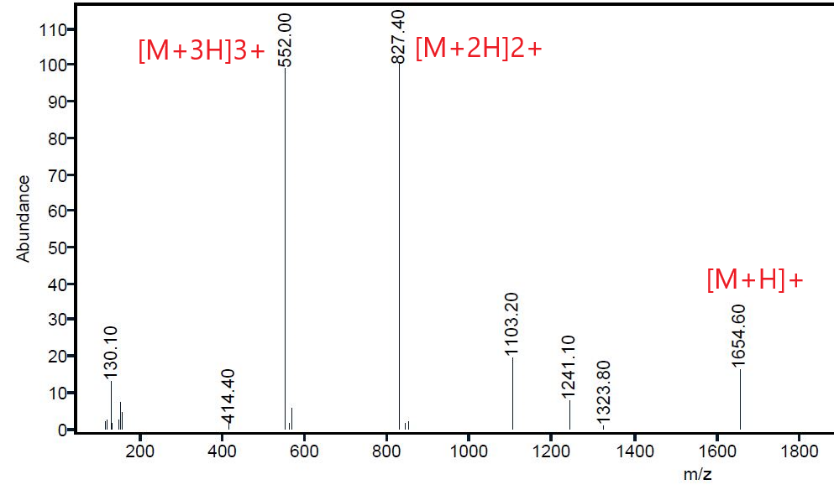


Figure S7: LC chromatogram and ESI-MS spectrum of N_3_-Py-DOTAGA-D-Phe-cyclo[Cys-Tyr-D-Trp-Lys-Thr-Cys]-Thr-OH (**10**).


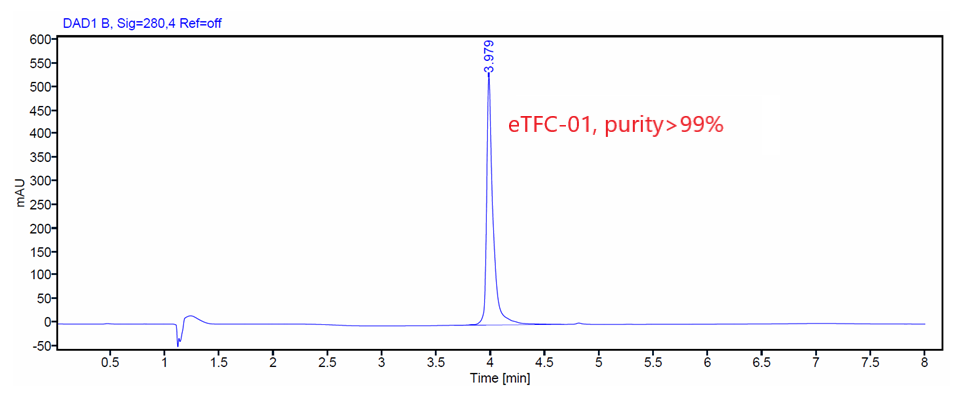


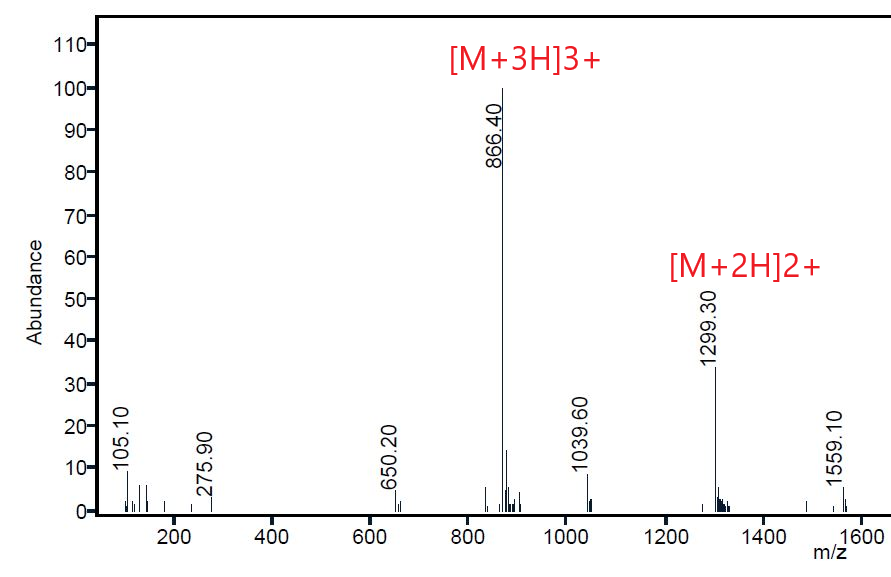


Figure S8: LC chromatogram and ESI-MS spectrum of **eTFC-01** (**11**).


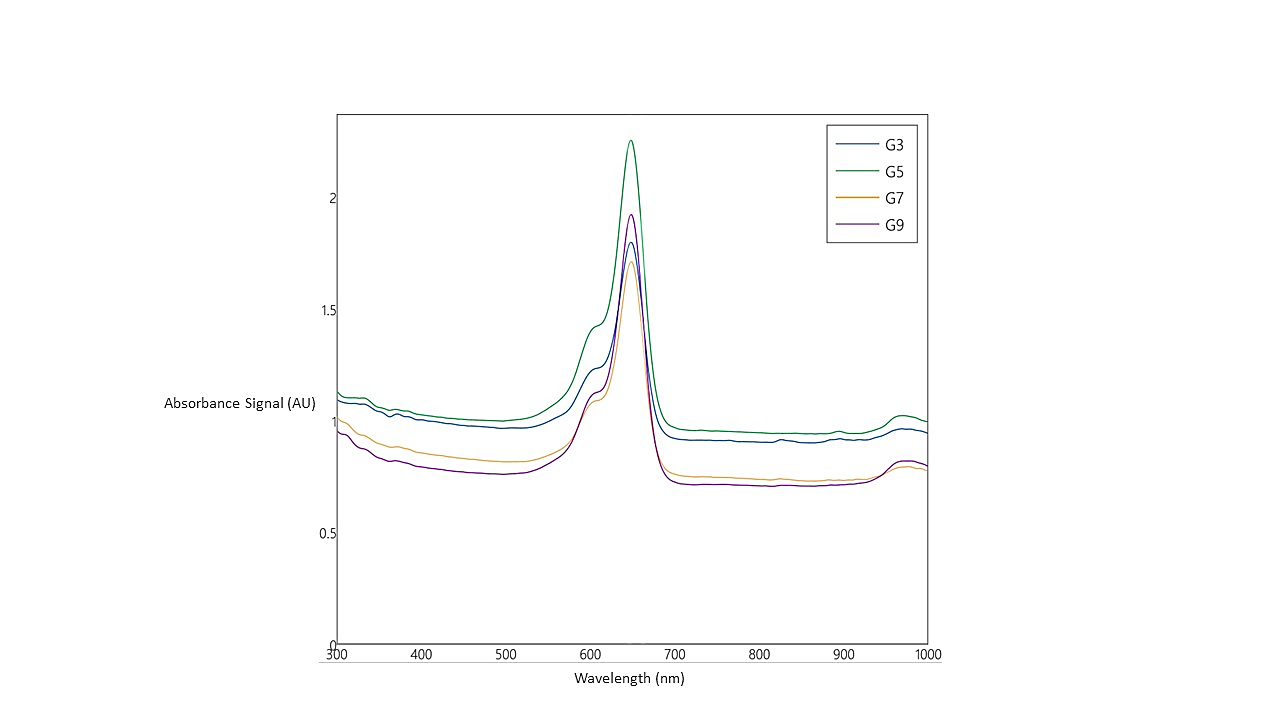


Figure S9: Absorbance spectrum of **eTFC-01** and Sulfo-Cy5 DBCO at two concentrations: 10^-4^ M (G5 and G9 for **eTFC-01** and Sulfo-Cy5 DBCO, respectively) and 10^-5^ M (G3 and G7 for **eTFC-01** and Sulfo-Cy5 DBCO, respectively)


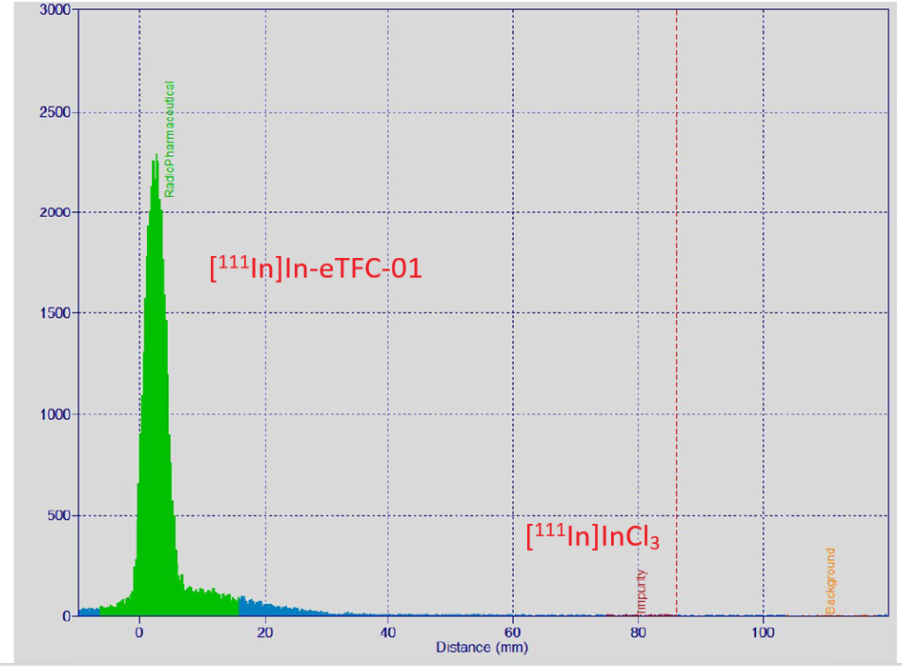


Figure S10: iTLC spectrum of [^111^In]In-**eTFC-01**.


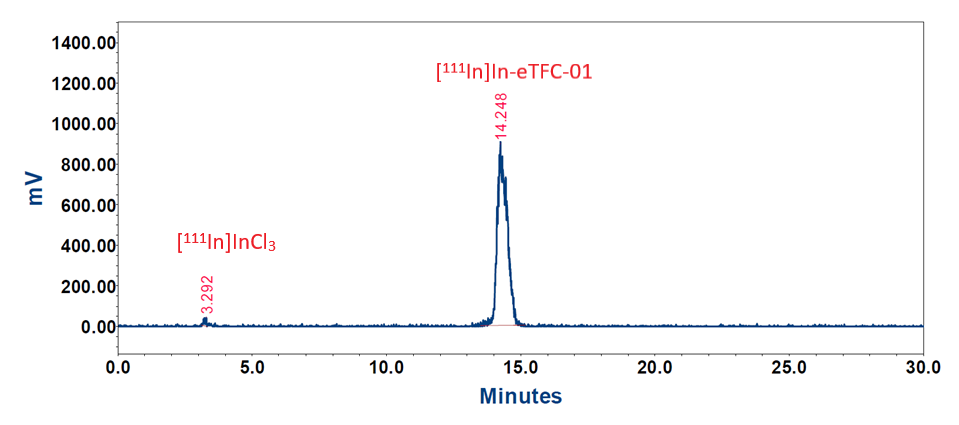


Figure S11: Radio-HPLC chromatogram of [^111^In]In-**eTFC-01***.*


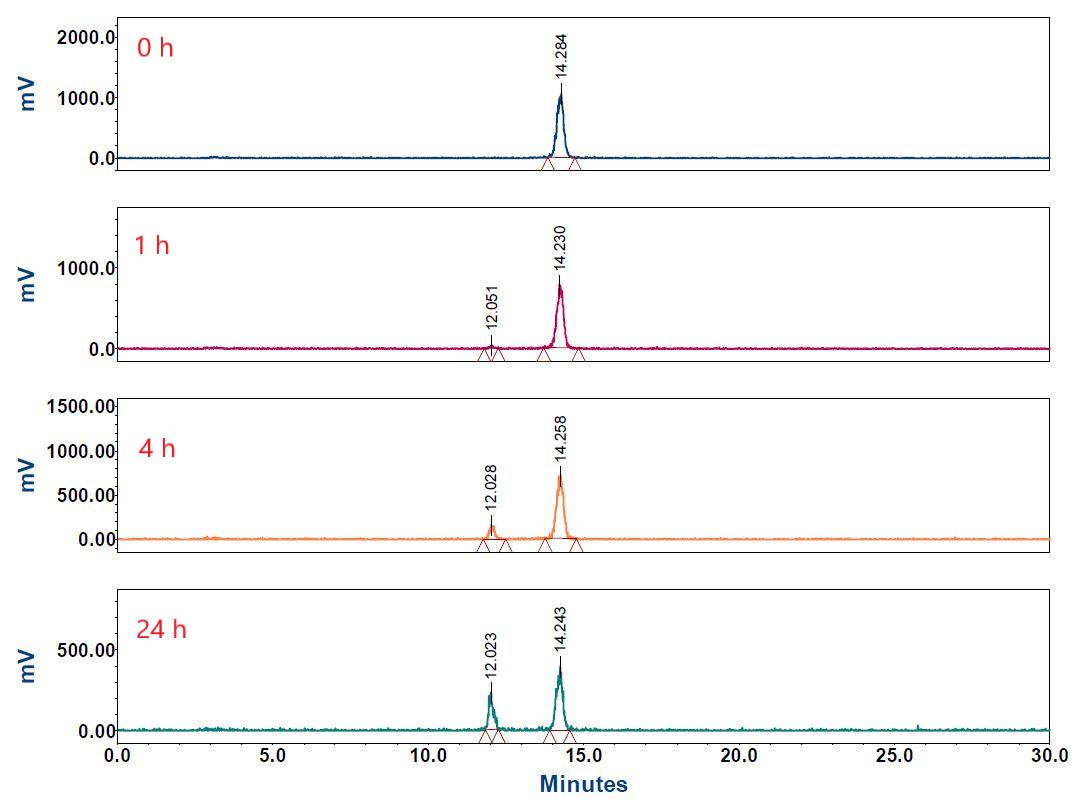


Figure S12: Stability of [^111^In]In-**eTFC-01** in mouse serum


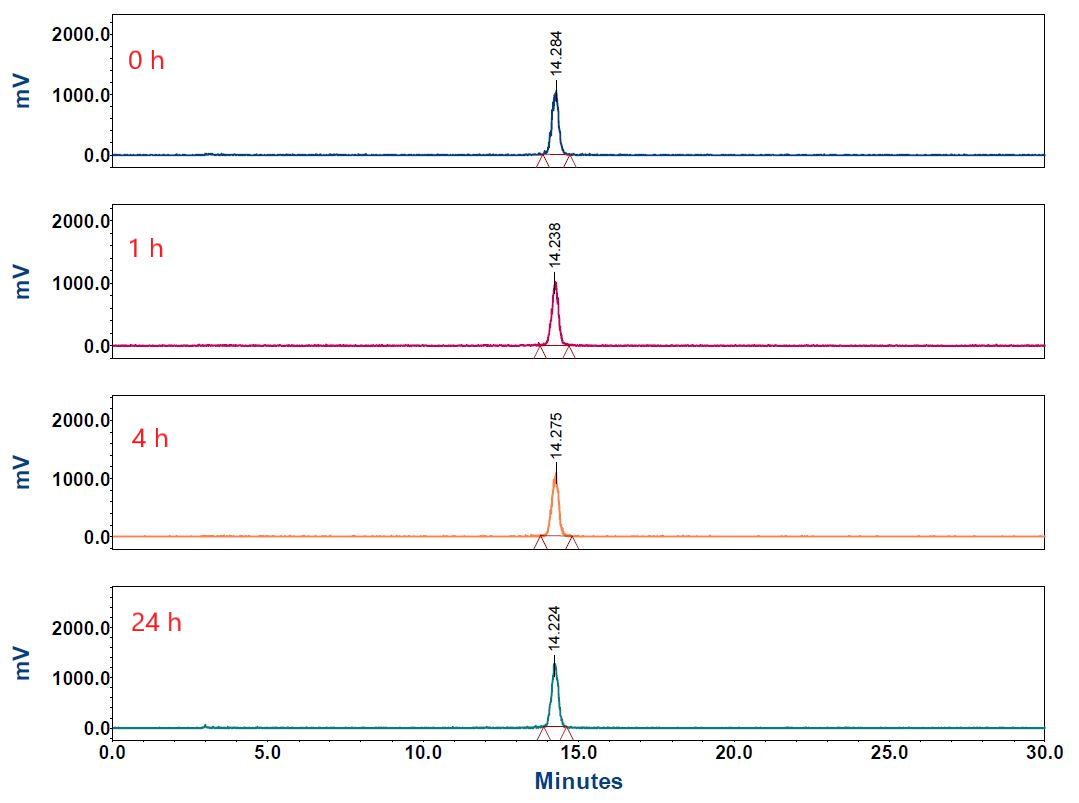


Figure S13: Stability of [^111^In]In-**eTFC-01** in PBS

Table S1: *Ex vivo* biodistribution data of [^111^In]In-DOTA-TATE at 1, 4, 4 h-block and 24 h post-injection (n = 4 mice/group). Data are represented as percentage of injected dose per gram of tissue (% ID/g).

| **Tissues** | **1 h** | **4 h** | **4 h block** | **24 h** |
| --- | --- | --- | --- | --- |
| Blood | 0.41 ± 0.14 | 0.04 ± 0.01 | 0.04 ± 0.02 | 0.04 ± 0.03 |
| Tumor | 5.55 ± 1.31 | 5.47 ± 0.30 | 0.17 ± 0.04 | 3.19 ± 0.29 |
| Heart | 0.16 ± 0.08 | 0.04 ± 0.00 | 0.05 ± 0.02 | 0.03 ± 0.00 |
| Lungs | 0.86 ± 0.09 | 0.52 ± 0.13 | 0.09 ± 0.02 | 0.43 ± 0.06 |
| Liver | 0.21 ± 0.03 | 0.16 ± 0.01 | 0.15 ± 0.08 | 0.08 ± 0.01 |
| Spleen | 0.22 ± 0.02 | 0.13 ± 0.02 | 0.11 ± 0.03 | 0.11 ± 0.01 |
| Stomach | 1.84 ± 0.18 | 1.23 ± 0.16 | 0.10 ± 0.03 | 0.51 ± 0.13 |
| Intestines | 0.74 ± 0.30 | 0.77 ± 0.24 | 0.57 ± 0.35 | 0.13 ± 0.01 |
| Pancreas | 2.90 ± 0.60 | 1.30 ± 0.10 | 0.07 ± 0.04 | 0.60 ± 0.05 |
| Kidneys | 12.19 ± 1.33 | 10.58 ± 0.39 | 7.75 ± 1.03 | 6.93 ± 1.02 |
| Muscle | 0.07 ± 0.04 | 0.02 ± 0.00 | 0.04 ± 0.03 | 0.01 ± 0.01 |
| Skin | 0.49 ± 0.26 | 0.29 ± 0.09 | 0.28 ± 0.13 | 0.13 ± 0.02 |
| Bones | 0.25 ± 0.14 | 0.13 ± 0.03 | 0.07 ± 0.02 | 0.14 ± 0.05 |

Table S2: *Ex vivo* biodistribution data of [^111^In]In-**eTFC-01** at 1, 4, 4 h-block and 24 h post-injection (n = 4 mice/group). Data are represented as percentage of injected dose per gram of tissue (% ID/g).

| **Tissues** | **1 h** | **4 h** | **4 h block** | **24 h** |
| --- | --- | --- | --- | --- |
| Blood | 6.96 ± 0.78 | 4.70 ± 0.28 | 4.11 ± 0.16 | 0.64 ± 0.11 |
| Tumor | 2.56 ± 0.38 | 3.04 ± 0.49 | 1.81 ± 0.14 | 2.21 ± 0.31 |
| Heart | 4.14 ± 0.76 | 2.50 ± 0.17 | 2.14 ± 0.46 | 0.74 ± 0.12 |
| Lungs | 8.07 ± 1.18 | 7.71 ± 0.87 | 6.05 ± 0.59 | 1.75 ± 0.19 |
| Liver | 8.88 ± 0.90 | 8.29 ± 0.81 | 10.61 ± 1.55 | 6.03 ± 1.14 |
| Spleen | 5.09 ± 0.72 | 3.58 ± 1.02 | 3.97 ± 0.90 | 2.03 ± 0.43 |
| Stomach | 2.66 ± 0.49 | 1.41 ± 0.19 | 0.43 ± 0.12 | 0.77 ± 0.22 |
| Intestines | 1.85 ± 0.24 | 1.85 ± 0.24 | 1.48 ± 0.13 | 0.44 ± 0.09 |
| Pancreas | 1.99 ± 0.26 | 1.28 ± 0.17 | 0.95 ± 0.23 | 0.64 ± 0.18 |
| Kidneys | 33.21 ± 3.27 | 36.56 ± 2.02 | 46.65 ± 6.34 | 30.60 ± 5.90 |
| Muscle | 1.07 ± 0.10 | 0.73 ± 0.08 | 0.67 ± 0.08 | 0.26 ± 0.05 |
| Skin | 5.73 ± 1.28 | 3.78 ± 0.35 | 3.61 ± 0.40 | 1.54 ± 0.35 |
| Bones | 2.29 ± 0.17 | 1.70 ± 0.23 | 1.53 ± 0.27 | 0.98 ± 0.21 |

Table S3: *Ex vivo* fluorescence measurements of [^111^In]In-**eTFC-01** at 1, 4, 4 h-block and 24 h post-injection (n = 4 mice/group). Data are represented as average radiant efficiency [10^10^ x (photons/second/cm^2^/steradian)/(mW/cm^2^)]

| **Tissues** | **1 h** | **4 h** | **4 h block** | **24 h** |
| --- | --- | --- | --- | --- |
| Tumor | 102.83 ± 21.91 | 120.63 ± 30.64 | 71.77 ± 5.44 | 121.00 ± 4.36 |
| Heart | 16.97 ± 2.27 | 16.41 ± 1.03 | 11.23 ± 2.49 | 7.06 ± 0.92 |
| Lungs | 82.87 ± 32.20 | 118.97 ± 16.52 | 54.37 ± 28.87 | 35.55 ± 12.14 |
| Liver | 188.67 ± 32.19 | 184.67 ± 48.95 | 156.33 ± 23.50 | 55.43 ± 11.24 |
| Spleen | 20.77 ± 5.03 | 16.32 ± 2.36 | 12.27 ± 0.83 | 10.61 ± 1.87 |
| Stomach | 59.77 ± 14.44 | 75.09 ± 20.13 | 35.47 ± 4.05 | 33.68 ± 6.86 |
| Intestines | 51.57 ± 4.37 | 61.26 ± 2.92 | 34.10 ± 8.23 | 18.90 ± 1.43 |
| Pancreas | 70.03 ± 8.54 | 31.80 ± 12.49 | 34.80 ± 6.97 | 24.68 ± 8.38 |
| Kidneys | 387.67 ± 102.07 | 613.23 ± 147.42 | 717.33 ± 37.54 | 448.00 ± 75.60 |
| Muscle | 19.83 ± 5.09 | 16.57 ± 1.63 | 17.17 ± 4.56 | 8.07 ± 0.91 |
| Skin | 84.73 ± 24.93 | 88.70 ± 19.09 | 108.13 ± 61.50 | 35.00 ± 14.33 |
| Bones | 23.30 ± 3.00 | 19.65 ± 10.74 | 17.13 ± 5.89 | 9.23 ± 1.40 |
